# Induction of oxidative metabolism by the p38α/MK2 pathway

**DOI:** 10.1038/s41598-017-11309-7

**Published:** 2017-09-12

**Authors:** Natalia Trempolec, Juan Pablo Muñoz, Konstantin Slobodnyuk, Silvia Marin, Marta Cascante, Antonio Zorzano, Angel R. Nebreda

**Affiliations:** 1 Institute for Research in Biomedicine (IRB Barcelona), Barcelona Institute of Science and Technology, 08028 Barcelona, Spain; 2 Department of Biochemistry and Molecular Biomedicine, Faculty of Biology, 08028 Barcelona, Spain; 3 CIBER de Diabetes y Enfermedades Metabólicas Asociadas (CIBERDEM), Instituto de Salud Carlos III, Madrid, Spain; 4 Institute of Biomedicine of the University of Barcelona (IBUB) and Research Associated Unit to CSIC, Barcelona, Spain; 5 ICREA, Pg. Lluís Companys 23, 08010 Barcelona, Spain

## Abstract

Adequate responses to environmental stresses are essential for cell survival. The regulation of cellular energetics that involves mitochondrial energy production and oxidative stress is central in the process of stress adaptation and response. The p38α signalling pathway plays a key role in the response to stress stimuli by orchestrating multiple cellular processes. However, prolonged activation of the p38α pathway results in impaired cell proliferation and can lead to cell death. Here we use a system to specifically activate p38α signalling and show that sustained activation of this pathway suffices to induce important metabolic changes, including high dependence on glucose for cell survival, increased consumption of glutamine, enhanced respiration rate and elevated production of mitochondrial reactive oxygen species (ROS). Moreover, we provide evidence that increased production of mitochondrial superoxide as a consequence of elevated mitochondria activity, contributes to the p38α reduced cell survival triggered by sustained p38α activation. We also show that the p38α-activated kinase MAPKAPK2 (MK2) plays an important role orchestrating the observed metabolic changes. Our results illustrate a new function of p38α signalling in the regulation of cellular metabolism, which may lead to cell death upon persistent activation of the pathway.

## Introduction

Proper responses to environmental stresses are critical for cell survival. Therefore, cells have developed sophisticated systems to receive and interpret stress signals. One of the pathways that plays an important role in the regulation of the stress response is orchestrated by the p38α serine-threonine protein kinase. p38α is a member of the mitogen-activated protein kinase (MAPK) family that is expressed in most tissues^1, 2^, and is normally activated by the dual-specificity MAPK kinases (MKKs) MKK3 or MKK6. The ability of p38α to phosphorylate a wide range of downstream substrates, including several transcriptional factors and other protein kinases, makes it an important regulator of cell proliferation, survival and differentiation, affecting multiple physiological processes^3, 4^. MAPKAPK-2 (also known as MK2) is one of the p38α substrates, which has been implicated in signalling events affecting the regulation of stress and inflammatory responses. MK2 can phosphorylate several proteins involved in transcriptional regulation, mRNA stability and other processes broadening the targets of the p38α pathway^5^.

The regulation of cell metabolism and mitochondrial energy production is central for cells to be able to adapt to stressful situations. A clear case of metabolic reprogramming is observed in cancer cells, which display important metabolic changes in order to support their elevated anabolic, energetic and redox demands^6, 7^. Based on these characteristics, new therapeutic approaches have been proposed by targeting mitochondrial function or using anti-glycolytic agents to overcome the resistance of cancer cells to conventional chemotherapy. Several signalling pathways and transcriptional regulators have been implicated in the modulation of cancer cell metabolism, with c-Myc, and PPARγ cofactor-1α (PGC1α) playing an important role in key biosynthetic processes and mitochondria generation^8–11^.

There is some evidence implicating p38α signalling in the regulation of metabolism in specific cell types, for example controlling glucose uptake in adipocytes and cardiac myocytes^12–14^, or gluconeogenesis in hepatocytes^15, 16^, but the role of p38α in cancer cell metabolism has not been investigated. Here we have investigated the effects of p38α activation on the metabolism of U2OS cancer cells. We show that sustained p38α activation increases nutrient consumption, with particularly high demands on glucose, and boosts mitochondrial efficiency, increasing mitochondrial mass and maximizing oxidative phosphorylation. This in turn elevates oxygen consumption resulting in the production of mitochondrial reactive oxygen species (ROS) that sensitize to cell death. Our results also indicate that MK2 plays a key role mediating the metabolic changes induced by p38α activation.

## Results

### Sustained activation of the p38α pathway triggers high dependence on glucose

**Figure 1:**
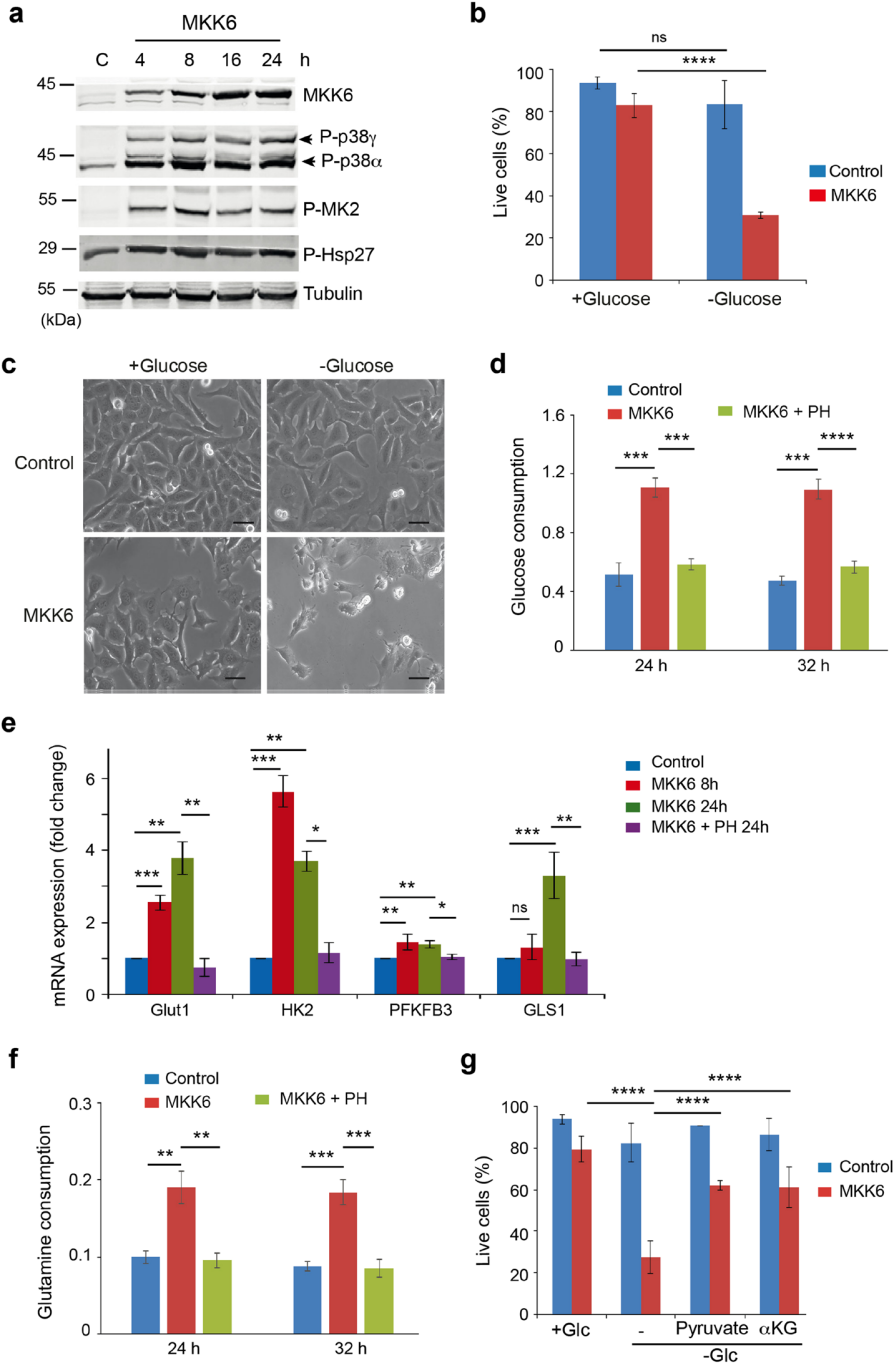
MKK6 expression makes cells highly dependent on glucose for survival. U2OS cells expressing a Tet-regulated construct were either mock treated (control) or treated with tetracycline for the indicated times to induce the expression of constitutively active MKK6. (**a**) Total cell lysates were analysed by immunoblotting using the indicated antibodies. Phosphorylated p38α and p38γ are indicated by arrowheads. The uncropped immunoblots are presented in Supplementary Fig. S8. (**b**) Control and MKK6 expressing cells were incubated in complete media or glucose free media for 24 h and cell survival was assayed using Annexin V/PI staining. Live cells were determined as the cell population Annexin V^−^ and PI^−^. (**c**) Morphology of cells cultured in complete or glucose free media for 24 h. Bars = 50 μm. (**d**) Glucose consumption by control, MKK6 expressing cells and MKK6 expressing cells treated with the p38α inhibitor PH797804 (PH) were measured at the indicated times and presented as μmol × 10^6^ cells^−1^ × h^−1^. (**e**) Control and MKK6 expressing cells were grown in the presence or absence of the p38α inhibitor PH797804 (PH) for the indicated times and the expression levels of mRNAs encoding glycolytic genes and GLS1 was analysed. Results are presented as fold change towards control. (**f**) Glutamine consumption was determined as in (**d**). (**g**) Control and MKK6 expressing cells were grown for 24 h in complete (+Glc) and glucose depleted media (−Glc) supplemented with 2 mM pyruvate or 1 mM dimethyl-2-ketoglutarate (αKG), as indicated, and cell survival was assayed using Annexin V/PI staining.

To better understand the effects triggered by activation of the p38α signalling pathway, we developed an inducible system to express MKK6DD, a constitutively active form of the specific p38 MAPK activator MKK6, under the control of the TET-ON promoter in U2OS cells. Induction of MKK6DD (hereafter referred to as MKK6) upon addition of tetracycline led to the detection of two bands that were recognized with an antibody specific for the phosphorylated (and active) form of p38 MAPKs (Fig. 1a). Using specific shRNAs, we identified the lower band as p38α and the upper band as p38γ (Supplementary Fig. S1a and b). MKK6 expression rapidly increased the phosphorylation of p38α, which correlated with enhanced phosphorylation of the p38α substrate MK2 and its downstream target Hsp27, whereas p38γ showed somewhat slower kinetics of activation (Fig. 1a). We found that MKK6 expression reduced U2OS cell proliferation, as previously reported^17^, and this effect was rescued by either chemical inhibition or downregulation of p38α but it was not affected by p38γ downregulation (Supplementary Fig. S1c).

The p38α pathway can be activated by multiple stresses, including nutrient deprivation^18^. Given the importance of glucose metabolism in cancer cells, we decided to investigate how direct activation of p38α signalling affected glucose metabolism in U2OS cells. We found that MKK6 expression led to high dependence on glucose, as shown by a dramatic decrease in cell viability under glucose-deprived conditions (Fig. 1b and c). The high dependence on glucose correlated with increased glucose uptake, detected 24 h after MKK6 expression, which was reversed upon inhibition of p38α using the chemical compound PH-797804 (Fig. 1d). Consistent with the above observations, MKK6 expression for 8 h induced the upregulation of mRNAs encoding the glucose transporter GLUT1, hexokinase 2 (HK2) and 6-phosphofructo-2-kinase (PFKFB3) (Fig. 1e). Additionally, 24 h after MKK6 expression, we detected increased expression of glutaminase-1 (GLS1), which generates glutamate from glutamine during the first step of glutamine metabolism (Fig. 1e). Indeed, p38α activation led also to enhanced glutamine consumption (Fig. 1f).

The activation of p38α did not seem to affect the tricarboxylic acid (TCA) reaction, as supplementation of glucose depleted media with dimethyl-2-ketoglutarate (αKG), a cell membrane-permeable precursor of key TCA intermediates, led to a significant increase in the survival of MKK6 expressing cells under glucose deprived conditions (Fig. 1g). Interestingly, cell death induced by glucose deprivation was also rescued by supplementation of the media with pyruvate (Fig. 1g), suggesting that p38α activation leads to changes in the cell metabolism in such a way that glucose becomes essential for mitochondrial metabolism.

### p38α activation increases glucose oxidation and fatty acid synthesis

**Figure 2:**
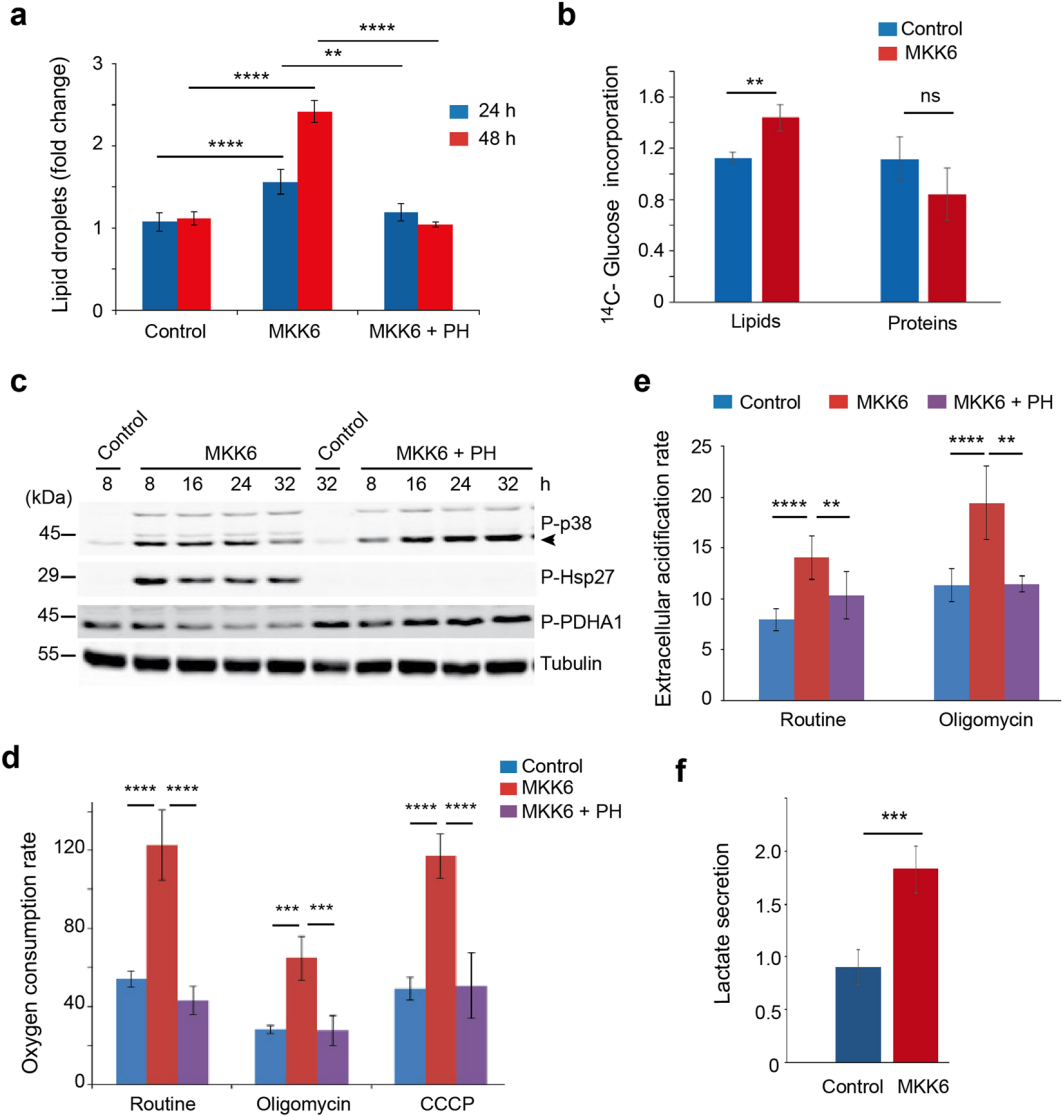
Metabolic changes triggered by MKK6 expression. U2OS cells expressing a Tet-regulated construct were either mock treated (control) or treated with tetracycline for the indicated times to induce the expression of constitutively active MKK6. (**a**) Lipid droplet content was quantified 24 h and 48 h after MKK6 induction in the presence or absence of the p38α inhibitor PH797804 (PH). (**b**) Incorporation of ^14^C-glucose into newly synthesized proteins and lipids was analysed 24 h after MKK6 induction, and is represented as fold change towards control. (**c**) Total lysates from control and MKK6 expressing cells in the presence or absence of PH were analysed by immunoblotting using the indicated antibodies. The uncropped immunoblots are presented in Supplementary Fig. S8. (**d**) The oxygen consumption rate was analysed 12 h after the induction of MKK6 expression in the presence or absence of PH. Results are presented as pmoles of O_2_ consumed per 10^5^ cells. (**e**) The extracellular acidification rate was measured 12 h after the induction of MKK6 in the presence or absence of PH. Results are presented as pmoles of secreted H^+^ per 10^5^ cells. (**f**) Lactate production was measured in control and MKK6 expressing cells at 32 h, and is presented as μmol × 10^6^ cells^−1^ × h^−1^.

Next, we analysed the impact of p38α activation on the utilization of substrates. We found that MKK6 expressing cells showed increased lipid droplet content, detected by Nile Red, which was impaired upon inhibition of p38α with PH-797804 (Fig. 2a and Supplementary Fig. S2). Lipid droplets can function as intracellular sites of neutral lipid storage, and are critical not only for lipid metabolism but also for energy homeostasis with their dysfunction being linked to multiple pathologies. Most of the carbon required for fatty acid synthesis comes from glucose delivered pyruvate, which after conversion to acetyl-CoA in mitochondria is excreted into the cytosol in the form of citrate for further lipid synthesis^10^. Using ^14^C-glucose we confirmed increased glucose incorporation into *de novo* synthesized lipids (Fig. 2b). The mitochondrial pyruvate dehydrogenase (PDH) complex plays a gatekeeper’s role in the regulation of carbon-flux by converting pyruvate into acetyl-CoA. Expression of MKK6 reduced the inhibitory phosphorylation of the E1α subunit of PDH (PDHA1), which was reversed by PH-797804, suggesting that p38α signalling positively regulates the activity of PDH leading to increased pyruvate oxidation (Fig. 2c). Consistent with this possibility, we detected that MKK6 expression increased the oxygen consumption rate (OCR) in basal (routine) conditions, which was mediated by p38α activation, based on its reversion by PH-797804 (Fig. 2d and Supplementary Fig. S3). The OCR increase was at least partly coupled to ATP production, since it was reduced in the presence of Oligomycin (Fig. 2d). Moreover, the enhanced metabolic rate correlated with an increased extracellular acidification rate (ECAR) (Fig. 2e), which predominately reflects the secretion of lactic acid after its conversion from pyruvate^19^. However, additional metabolic processes, such as CO_2_ production by the TCA cycle, can also lead to ECAR changes^20^. We also found that MKK6 expressing cells secreted more lactate (Fig. 2f), suggesting that sustained activation of p38α leads to increased glycolysis in addition to enhanced glucose oxidation. Recently, it has been shown that tetracyclines, even at low concentration, can induce mitochondrial proteotoxic stress, leading to changes in mitochondrial dynamics and function^21^. To rule out the possibility that the effects mentioned above could be induced by tetracycline treatment independently of MKK6 expression, we used parental U2OS cells without the inducible system to express MKK6. We found that treatment of these cells with tetracycline neither activated the p38α pathway (Supplementary Fig. S4a) nor increased OCR, which if anything slightly decreased in both basal and maximum respiration rate (Supplementary Fig. S4b). These results support the positive effect of p38α signalling on mitochondria functionality.

### p38α activation induces mitochondrial biogenesis

**Figure 3:**
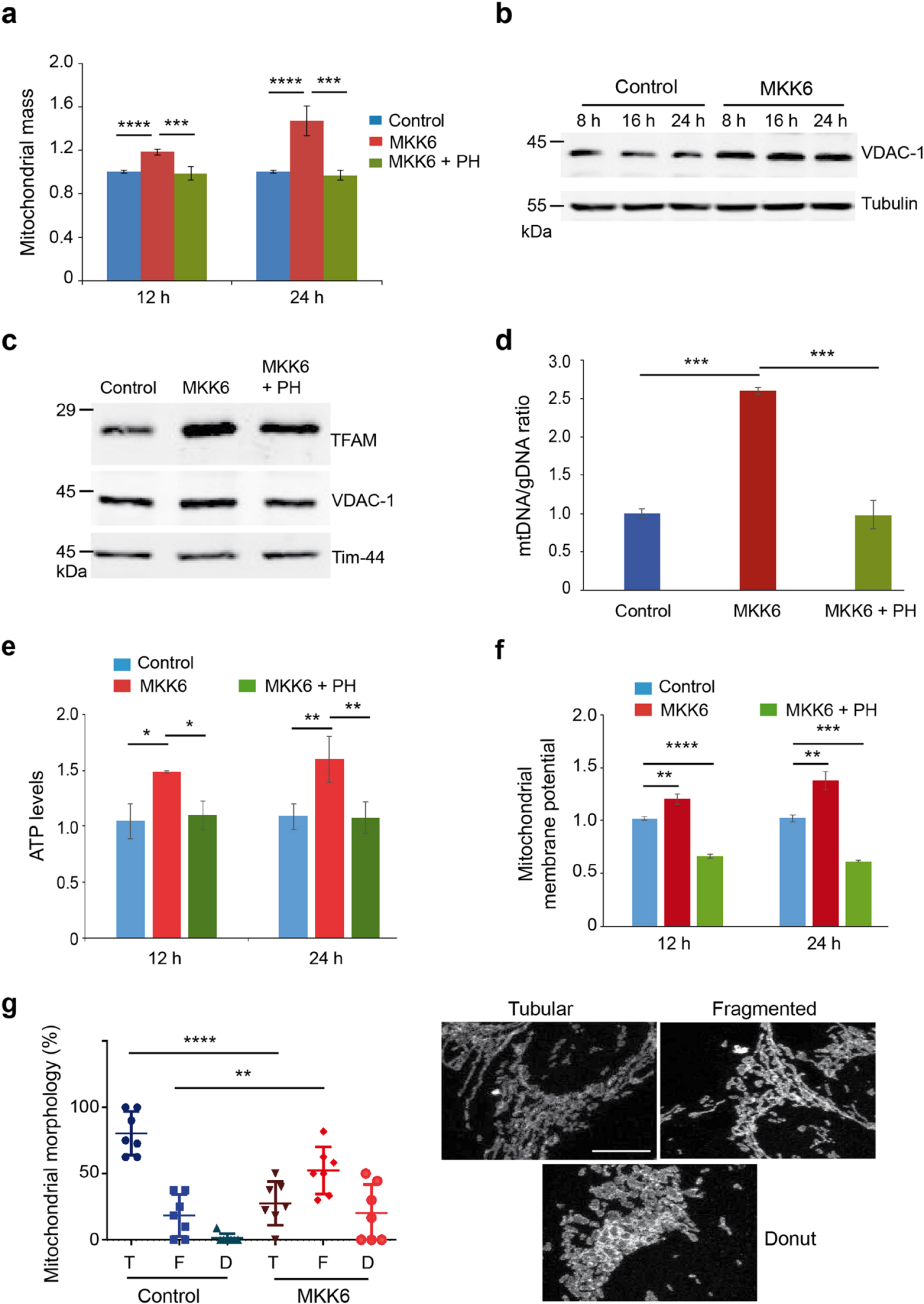
MKK6 expression affects mitochondria function. U2OS cells expressing a Tet-regulated construct were either mock treated (control) or treated with tetracycline for the indicated times to induce the expression of constitutively active MKK6. (**a**) Mitochondrial mass in control and MKK6 expressing cells was measured using MitoTracker Deep Red at the indicated times, and is represented as fold change versus the control. (**b**) Total lysates from control and MKK6 expressing cells were analysed by immunoblotting. (**c**) Mitochondria were extracted from control and MKK6 expressing cells in the presence or absence of the p38α inhibitor PH797804 (PH) for 24 h. Expression of the indicated mitochondrial proteins was analysed by immunoblotting. The uncropped immunoblots are presented in Supplementary Fig. S8. (**d**) The ratio of mitochondrial DNA (mtDNA) versus genomic DNA (gDNA) was analysed in control and MKK6 expressing cells in the presence or absence of PH for 24 h. (**e**) ATP levels were analysed at the indicated times after MKK6 induction in the presence or absence PH and were normalized towards the cell number. Values were standardised towards control cells. (**f**) Mitochondrial membrane potential in control and MKK6-expressing cells in the presence or absence of PH was measured at 12 and 24 h. (**g**) Quantification of control and MKK6 expressing cells at 24 h that contain tubular (T), fragmented (F) and donut (D) mitochondria visualized with Tom 20 staining. Bar = 10 μM.

Increased mitochondrial respiration can be due to changes in either the mitochondrial mass or the mitochondrial functionality. Staining with Mitotracker Deep Red showed that MKK6 induction increased the mitochondrial compartment and this effect was reversed by p38α inhibition (Fig. 3a), but was not observed in tetracycline-treated U2OS cells that do not express MKK6 (Supplementary Fig. S4c). Consistent with the idea that p38α activation induced mitochondrial biogenesis, we observed the p38α-induced upregulation of Voltage-dependent anion-selective channel protein 1 (VDAC1), a porin located in the outer membrane of mitochondria (Fig. 3b). Immunoblot analysis of isolated mitochondria further supported the enhanced mitochondria biogenesis in MKK6-expressing cells, based on the accumulation of the mitochondrial transcription factor A (TFAM), which controls transcription of mitochondrial DNA encoded genes as well as DNA replication during biogenesis (Fig. 3c). This increased presence of TFAM in mitochondria agrees with the increased mitochondrial DNA copy number normalized by genomic DNA copy number induced by MKK6 expression (Fig. 3d). These changes correlated with increased mitochondrial functionality, as determined both by elevated ATP production (Fig. 3e) and by enhanced mitochondrial membrane potential (Fig. 3f). Two factors involved in canonical mitochondria biogenesis are c-Myc and PGC1α^22–24^. We found that p38α activation increased the expression of both (Supplementary Fig. S5a and b), but knocking down c-Myc or PGC1α did not affect the increased mitochondrial mass observed in MKK6 expressing cells (Supplementary Fig. S5c–f).

Correct mitochondrial dynamics is essential for cell survival and is subjected to rapid changes in response to external insults and metabolic status^25^. Curiously, we observed that MKK6 expression induced the fragmentation of mitochondria resulting in reduced number of tubular structures and appearance of more rounded structures (Fig. 3g).

### Mitochondrial superoxide production contributes to p38α-induced cell death

**Figure 4:**
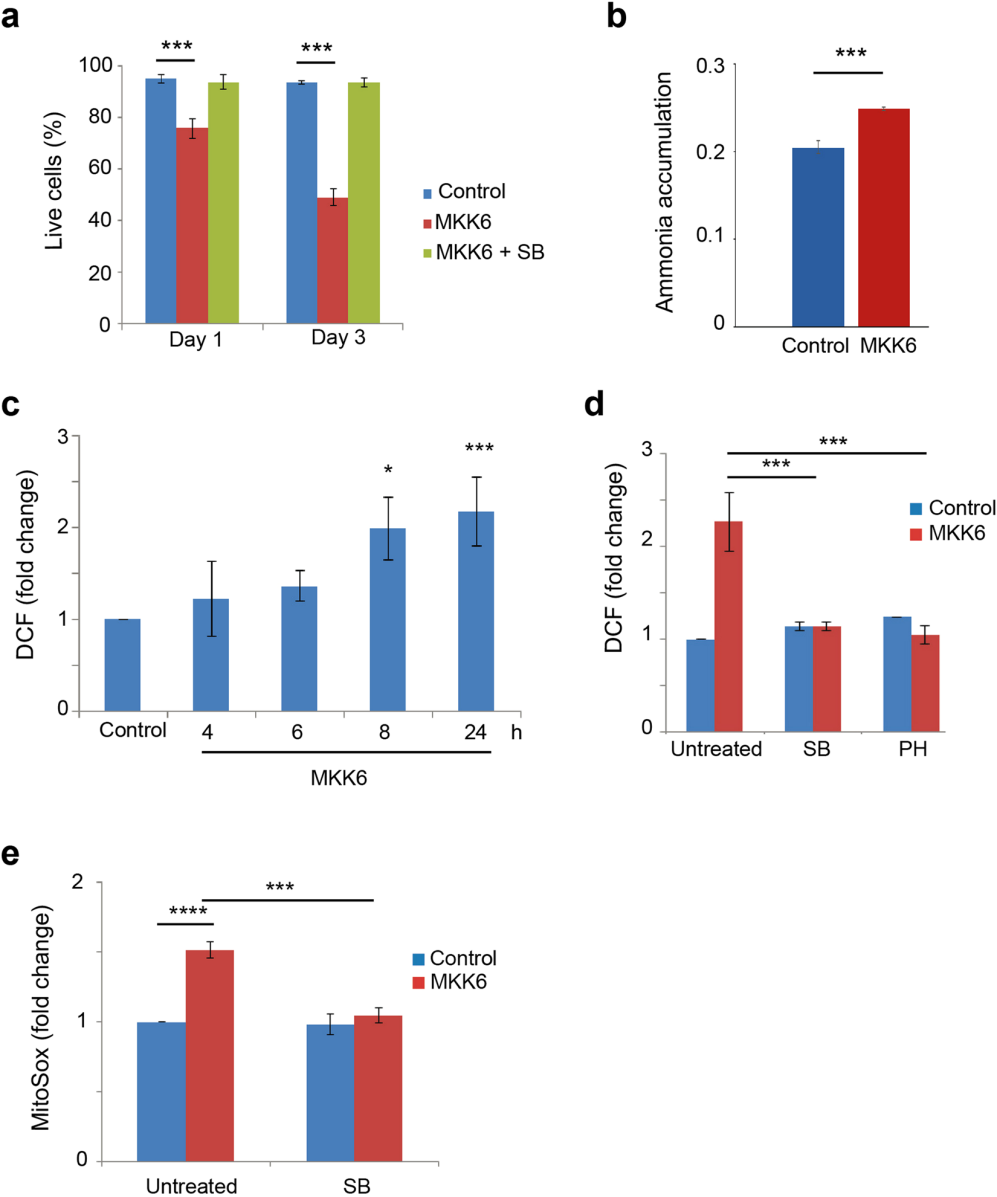
Increased mitochondrial ROS contributes to cell death induced by MKK6 expression. U2OS cells expressing a Tet-regulated construct were either mock treated (control) or treated with tetracycline for the indicated times to induce the expression of constitutively active MKK6. (**a**) Cell survival was assayed at the indicated times using Annexin V/PI staining. Live cells were determined as the cell population Annexin V^−^ and PI^−^. (**b**) Intracellular ammonia accumulation was measured in control and MKK6 expressing cells at 32 h and is presented as nmol / 5 × 10^4^ cells. (**c**) Total ROS levels were analysed at the indicated times after MKK6 induction using the DCFH-DA probe. Values are presented as fold change of mean fluorescence of DCF versus the control cells. (**d**) ROS levels in control and MKK6 expressing cells treated for 24 h with the p38α inhibitors PH797804 (PH) or SB203580 (SB) were analysed using the DCFH-DA probe. Values are presented as fold change in the mean fluorescence of DCF. (**e**) Mitochondrial ROS levels in control and MKK6 expressing cells either mock-treated or treated with SB were evaluated at 24 h using the MitoSox probe. Values are represented as fold change in the mean fluoresce of MitoSox normalized towards control cells.

Mitochondrial fission is linked to apoptosis^26^. We found that expression of MKK6 resulted in reduced cell survival, which started to be detected at 24 h by Annexin V and PI staining, and was reversed by p38α inhibition (Fig. 4a and Supplementary Fig. S6a). Further analysis showed that treatment with the caspase inhibitors Z-VAD or Q-VD partially but significantly prevented the decline in live cell population triggered by MKK6 expression (Supplementary Fig. S6b). These results suggested that MKK6 expression probably induced a combination of different types of cell death, mediated by both caspase-dependent and independent events. On the other hand, treatment with the necrosis inhibitor Nec-1 did not affect the cell death triggered by MKK6 expression (Supplementary Fig. S6c).

Our results indicated that the p38α-induced metabolic reprogramming was already detected at 12 h, as determined by increased cell respiration, mitochondrial biomass and increased glucose consumption, which therefore preceded cell death that started to be detected at 24 h after p38α activation. The accelerated cell metabolism induced by expression of MKK6 also led to the enhanced generation of metabolic side products such as lactate (see Fig. 2f above) and ammonium (Fig. 4b), as well increased generation in ROS (Fig. 4c). The ROS increase required activation of p38α, as it was reversed by chemical inhibition of p38α (Fig. 4d) or by downregulation of p38α but it was not affected by p38γ downregulation (Supplementary Fig. S1d). Using the mitochondrial-specific probe MitoSox, we also detected increased mitochondrial ROS after MKK6 expression, which was reversed by the inhibition of p38α (Fig. 4e). However, consistent with the idea that p38α-induced metabolic changes precede cell death, the caspase inhibitor Z-VAD did not affect the MKK6-induced mitochondrial ROS increase (Supplementary Fig. S6d). Importantly, neither mitochondrial ROS nor cell survival levels were affected in tetracycline-treated U2OS cells that do not express MKK6 (Supplementary Fig. S4d and e).

**Figure 5:**
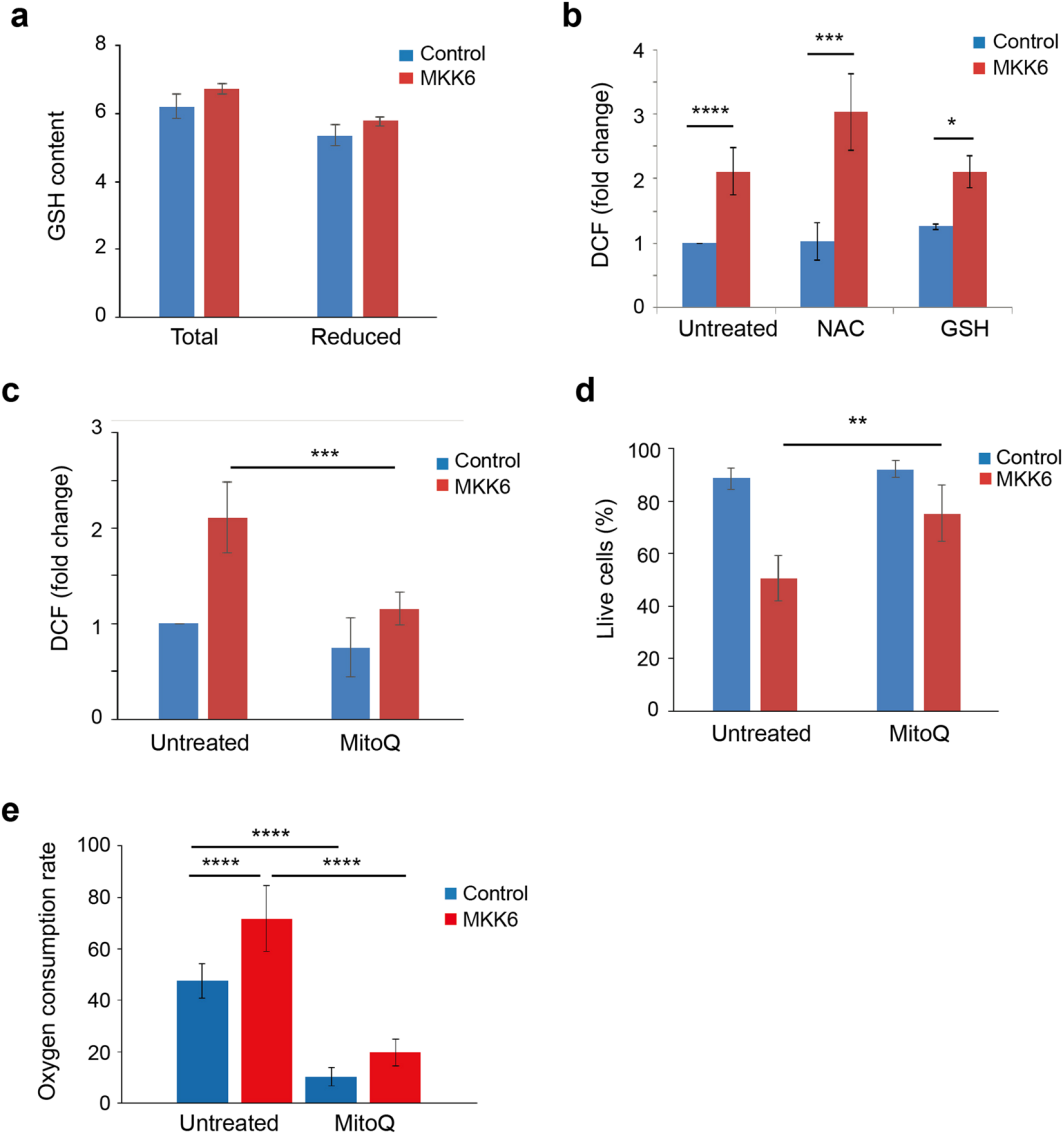
MKK6 expression accelerates cell metabolism. U2OS cells expressing a Tet-regulated construct were either mock treated (control) or treated with tetracycline for the indicated times to induce the expression of constitutively active MKK6. (**a**) GSH content (total and reduced) were analysed in control and MKK6 expressing cells at 24 h and presented as μmol/10^3^ cells. (**b**) ROS levels in control and MKK6 expressing cells treated with NAC (5 mM) and GSH (5 mM) were analysed at 24 h after MKK6 induction using the DCFH-DA probe. (**c**) ROS levels in control and MKK6 expressing cells treated with MitoQ (500 nM) were analysed at 24 h using the DCFH-DA probe. (**d**) Control and MKK6 expressing cells were treated with MitoQ (500 nM) for 2 days and cell survival was analysed by Annexin V/PI staining. Data were taken from 3 independent experiments. (**e**) The oxygen consumption rate was analysed 24 h after the induction of MKK6 expression in routine conditions and after addition of MitoQ (500 nM), as indicated. Results are presented as pmoles of O_2_ consumed per 10^5^ cells.

The intracellular antioxidant system is key to maintain the redox balance in the cell. A very important intracellular antioxidant is GSH that detoxifies H_2_O_2_ via GSH peroxidases giving rise to oxidized GSH (GSSG), which is then reduced into GSH by GSH reductase using NADPH as the electron donor. We found that MKK6 expression did not affect the levels of total or reduced GSH (Fig. 5a), suggesting that ROS did not increase due to the declined efficiency of the intracellular antioxidant system. This result was confirmed by treatment of MKK6 expressing cells with canonical antioxidants, such as NAC (N-acetyl cysteine) and GSH, which failed to reverse the ROS increase (Fig. 5b). On the other hand, the mitochondria-targeted antioxidant MitoQ^27^ decreased ROS induction by MKK6 (Fig. 5c). Interestingly, MitoQ substantially rescued the cell death triggered by MKK6 expression, suggesting that mitochondrial ROS contributes to the process (Fig. 5d). In order to investigate if superoxide production was associated with mitochondrial activity, we analysed whether MitoQ affected the OCR of MKK6 expressing cells. Addition of MitoQ to the cellular media has been shown to reduce oxygen consumption, which may be mediated by the inhibition of complex I of mitochondria independently of its antioxidant function^28, 29^. Accordingly, we found that incubation with MitoQ for 24 h decreased OCR in both control and MKK6 expressing cells (Fig. 5e), suggesting that superoxide generated due to increased activation of the respiratory chain leads to cell death.

### Metabolic changes induced by p38α activation are mediated by MK2

**Figure 6:**
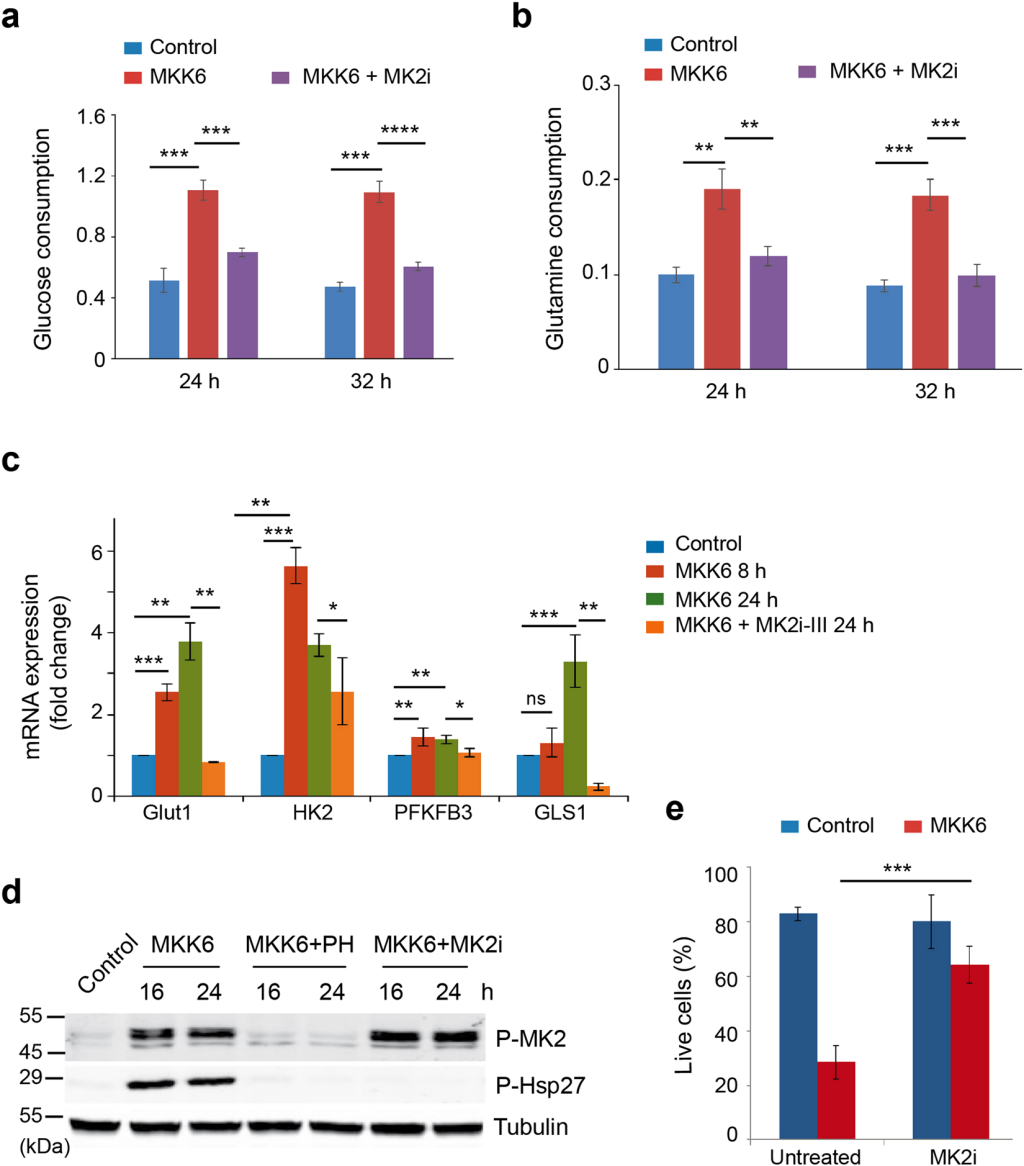
MK2 mediates the MKK6-induced metabolic changes. U2OS cells expressing a Tet-regulated construct were either mock treated (control) or treated with tetracycline for the indicated times to induce the expression of constitutively active MKK6. (**a** and **b**) Glucose consumption (**a**) and glutamine consumption (**b**) by control, MKK6 expressing cells and MKK6 expressing cells treated with the MK2 inhibitor PF3644022 (MK2i) were measured at the indicated times and presented as μmol × 10^6^ cells^−1^ × h^−1^. These samples were processed in parallel and using the same control and MKK6 expressing cells as the samples presented in Fig. 1d and f. (**c**) The expression levels of mRNAs encoding proteins involved in glycolysis and glutaminolysis were determined by qRT-PCR after MKK6 induction in the presence or absence of the MK2 inhibitor III (MK2i-III) for the indicated times. (**d**) Total cell lysates were prepared from control and MKK6 expressing cells in the presence or absence of the p38α inhibitor PH797804 (PH) or the MK2 inhibitor PF3644022 (MK2i), and were analysed by immunoblotting using the indicated antibodies. The uncropped immunoblots are presented in Supplementary Fig. S8. (**e**) Control and MKK6 expressing cells were grown in glucose-deprived conditions in the presence or absence of MK2i for 24 h, and cell survival was assayed using Annexin V/PI staining. Live cells were determined as the cell population Annexin V^−^ and PI^−^.

The protein kinase MK2 is a well-known substrate of p38α, whose activation was induced upon MKK6 expression (Fig. 1a). Little is known on the implication of MK2 in cell metabolism, although MK2 has been reported to mediate the stress-induced increase in glycolysis by upregulation of PFKFB3^30^. We decided to study the implication of MK2 in the metabolic changes induced by p38α activation. First, we found that a chemical inhibitor of MK2 reversed the increase in glucose and glutamine uptake induced by MKK6 expression (Fig. 6a and b), which correlated with reduced expression of GLUT1, PFKFB3 and GLS1 (Fig. 6c). Importantly, we confirmed that the chemical inhibitor impaired MK2 activity, as shown by the lack of Hsp27 phosphorylation, but did not affect p38 MAPK activity, as shown by the phosphorylation of MK2 itself (Fig. 6d). Interestingly, inhibition of MK2 reduced the cell death triggered by glucose deprivation in MKK6-expressing cells (Fig. 6e), supporting that metabolic changes mediated by MK2 sensitize the MKK6-expressing cells to the absence of glucose.

**Figure 7:**
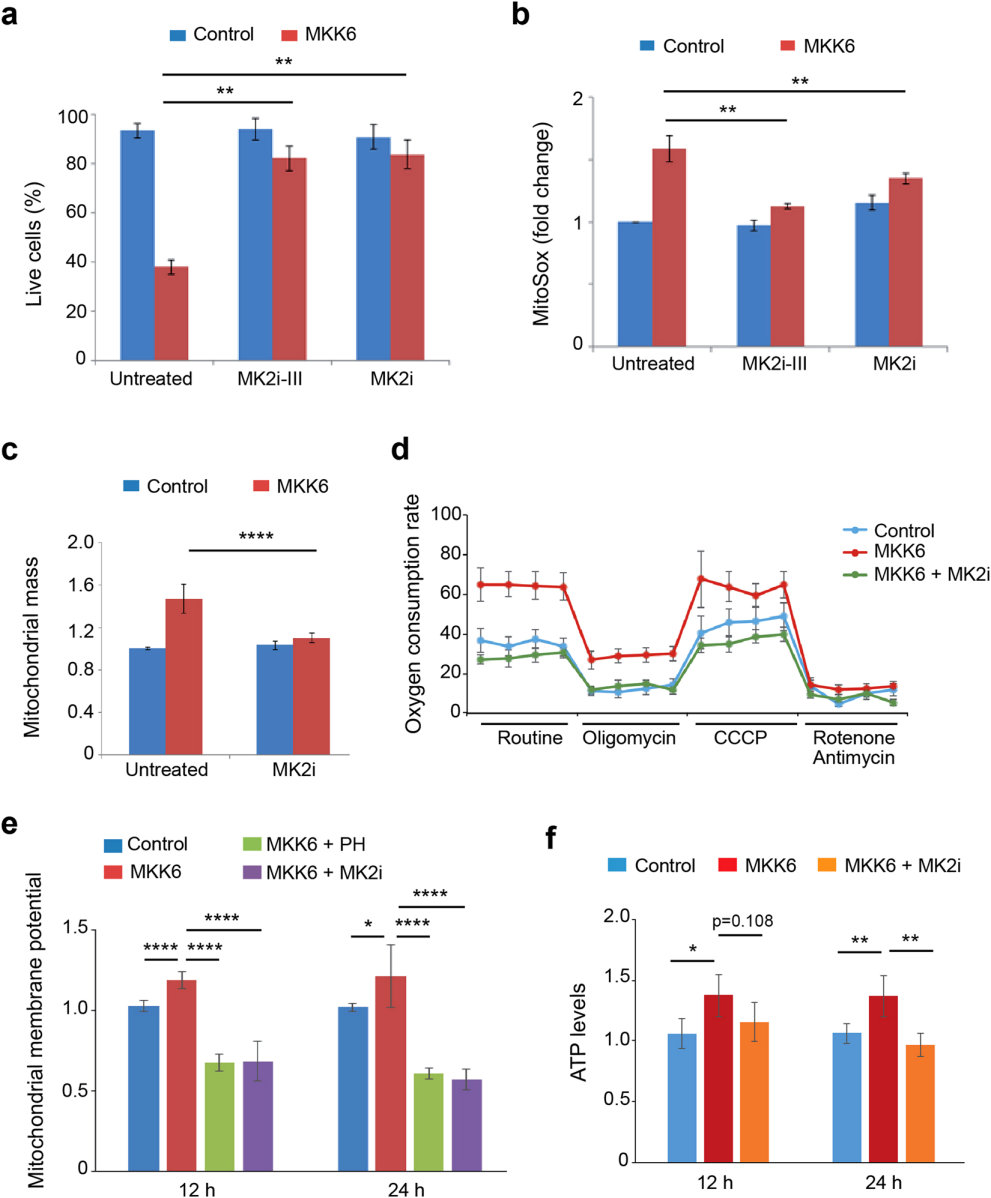
MK2 mediates changes in mitochondrial function induced by MKK6 expression. U2OS cells expressing a Tet-regulated construct were either mock treated (control) or treated with tetracycline for the indicated times to induce the expression of constitutively active MKK6. (**a**) Control and MKK6 expressing cells were grown in complete medium in the presence or absence of the MK2 inhibitors PF3644022 (MK2i) or MK2 inhibitor III (MK2i-III) for 3 days, and cell survival was assayed using Annexin V/PI staining. Live cells were determined as the cell population Annexin V^−^ and PI^−^. (**b**) Control and MKK6 expressing cells were incubated with MK2i or MK2i-III for 24 h, and mitochondrial ROS levels were analysed using the MitoSox probe. Values are represented as fold change in the mean fluorescence of MitoSox normalized towards control. (**c**) Control and MKK6 expressing cells were incubated with MK2i for 24 h and mitochondrial mass was analysed by Mitotracker Deep Red. Values are represented as fold change versus the control. (**d**) The oxygen consumption rate was analysed 24 h after the induction of MKK6 expression in the presence or absence of MK2i. Results are presented as pmoles of consumed O_2_ per 10^5^ cells. (**e**) Mitochondrial membrane potential in control and MKK6 expressing cells in the presence or absence of PH or MK2i was measured at the indicated times. (**f**) ATP levels were analysed at the indicated times after MKK6 induction in the presence or absence of MK2i, and were normalized towards the cell number.

We also found that inhibition of MK2 using two different chemical compounds significantly improved the survival of cells in response to MKK6 expression (Fig. 7a). Thus, we investigated the implication of MK2 in the increased oxidative metabolism that correlated with reduced viability of MKK6 expressing cells. Experiments using MK2 inhibitors revealed that the MKK6-induced mitochondrial ROS increase (Fig. 7b), as well as the enhanced mitochondrial mass (Fig. 7c) and oxygen consumption rate (Fig. 7d) were all mediated by MK2. Additionally, we found that MK2 activation was involved in the mitochondrial proton motive force, as MK2 inhibition reversed the increased mitochondria membrane potential (Fig. 7e) and elevated ATP production induced by MKK6 expression (Fig. 7f). Interestingly, we found that in contrast to MK2, chemical inhibition of MNK and MSK, two other protein kinases that can be directly activated by p38α^2^, did not affect cell death induced by MKK6 expression (Supplementary Fig. S7a and b). In agreement with the idea that p38α-induced mitochondrial dysfunction and enhanced ROS production were related to the observed cell death, we found that inhibition of MNK or MSK signalling reduced neither the mitochondrial mass increase nor the production of mitochondrial ROS induced by MKK6 expression (Supplementary Fig. S7c and d). Taken together, our results provide evidence for a novel role of the p38α pathway in the regulation of the mitochondrial mass and mitochondrial dependent ROS production, which is mainly mediated by MK2 activation.

## Discussion

Cells exposed to environmental stresses require signalling flexibility to generate the responses necessary to recover from insults. However, if the stress persists or inadequate stimulation or repression of particular signalling pathways occurs, cell death programs are often activated. The p38α pathway mediates intracellular signalling triggered by a variety of stimuli, which impinge on functions such as cell proliferation, differentiation and survival^2^. The different outcomes of p38α signalling can be due to the extent of pathway activity, with sustained p38α activation more likely to result in cell death, whereas transient or low levels of p38α activity are usually associated with homeostatic functions and cell survival^2, 31, 32^.

We have used a cellular system based on inducible MKK6 expression to specifically activate the p38 MAPK pathway and recapitulate potential pathological states associated with p38α hyper-activation. Our studies show that sustained activation of p38α in U2OS cancer cells leads to a striking dependence on glucose for cell survival, which correlates with increased glucose consumption and glycolysis rate. Altered glucose metabolism is an important feature of cancer cells, which metabolize glucose mainly through glycolysis, with mitochondria being reprogrammed for macromolecular synthesis^10, 11^. Enhanced glucose consumption triggered by p38α activation, can be due to increased demand for glycolytic intermediates, used for fatty acids and nucleotide synthesis as well as for protein acetylation. Increased flux of pyruvate into the essential glycolytic intermediate acetyl-CoA was supported by the increased PDH activity and the elevation in glucose-dependent lipid content observed upon p38α activation. Our results showing that addition of pyruvate allows cells with hyperactive p38α to survive in glucose-free medium, indicate that these cells are highly dependent on pyruvate for their anaplerotic reactions.

The metabolic boost observed upon sustained p38α activation, led us to explore the effect on mitochondrial metabolism. Activation of p38α suffices to induce oxidative phosphorylation, as determined by the elevated OCR, which was accompanied by increased mitochondrial mass. Changes in the mitochondria content can be due either to mitochondrial biogenesis or to defects in mitophagy, and we provide evidence that p38α activation increases mitochondrial biogenesis. Additionally, we show that cells with sustained p38α activity possess functional mitochondria, exhibiting increased mitochondrial membrane potential, ATP production and OCR. These changes are accompanied by increased expression of PGC1α, a key regulator of mitochondrial biogenesis, which can be positively regulated by p38α both at transcriptional and post-transcriptional levels^33, 34^. However, PGC1α downregulation does not affect the increase in mitochondrial biogenesis induced by p38α signalling, suggesting the implication of other p38α-regulated factors.

The regulation of metabolism and cell death are interconnected at multiple levels, with several proteins that control cell death being also implicated in metabolic regulation^35^. Increased nutrient uptake and glucose oxidation are in principle beneficial for highly proliferative cells, but when raised above certain limits, can cause detrimental effects due to the accumulation of side metabolites such as mitochondrial ROS. The mitochondrial electron transport chain, which sets up the proton motive force, is one of the most important sources for ATP and intracellular ROS production^36, 37^. Our results indicate that increased OCR and mitochondrial ROS production contribute to cell death induced by sustained p38α activation, based on the rescue observed using the mitochondrial specific antioxidant MitoQ^38^. Interestingly, this resembles the effect of dichloroacetic acid (DCA), a chemical inhibitor of PDH that enhances oxidative phosphorylation resulting in increased mitochondrial ROS and mitochondria-dependent apoptosis^39^. The reduced metabolic flexibility triggered by sustained activation of the p38α pathway might contribute to the high dependence on glucose for the survival of these cells.

MK2 is a well-established substrate of p38α involved in multiple cellular processes^5^. We found that MK2 plays an important role mediating the p38α triggered metabolic changes that lead to increased glucose consumption and reduced cell viability. Additionally, we show a new role of MK2 in increasing mitochondrial mass and activity. MK2 can phosphorylate the transcription factor CREB^40^, which has been implicated in the regulation of mitochondrial function^41^. Therefore, it is possible that the MKK6 induced changes in mitochondrial mass and functionality could be mediated by the MK2/CREB axis.

In summary, we provide evidence for a novel role of the p38α pathway increasing both ATP coupled and uncoupled respiration. The effects are triggered by specific activation of p38α signalling, which probably affects cell metabolism in a pleiotropic manner. The metabolic boost observed upon sustained p38α activation is probably beneficial for the cell in the early stages as it increases energy input and generates building blocks. However, sustained p38α activation beyond a certain threshold eventually leads to mitochondria dysfunction and reduced cell viability, emphasizing the importance of negative feed-back loops to restrain the extent of signalling pathway activation for proper cell homeostasis. Our results suggest that deregulated p38α signalling might contribute to human pathologies such as ageing and neurodegenerative disorders that are associated with impaired mitochondrial functions and ROS balance^42, 43^. Of note, increased OCR and ROS production induced by interfering with PDH activity has been shown to decrease tumour growth^39^, suggesting that sustained p38α activation could be beneficial to treat highly proliferative tumours.

## Methods

### Cell culture

U2OS cells (purchased from ATCC) were cultured in Dulbecco’s modified Eagle medium (DMEM, Sigma, D5796) supplemented with 10% fetal bovine serum (FBS, Thermo Scientific, E6541L), 2 mM L-Glutamine (LabClinics, M11–004) and 100 μg/ml penicillin-streptomycin (LabClinics, P11-010). For the inhibition of p38α, we used 1 μM PH-797804 (Selleckchem, S2726) or 10 μM SB203580 (Axon MedChem); both compounds can also inhibit p38β, although PH-797804 has been reported to preferentially inhibit p38α^44^. MK2 was inhibited using 10 μM MK-2 Inhibitor III (Calbiochem) or 10 μM PF 3644022 (Sigma, PZ0188), whereas MSK activity was inhibited using 5 μM SB 747651 (Axon MedChem), and MNK activity was inhibited using 10 μM CGP-57380 (Sigma). MitoQ (kind gift from M. Murphy, Cambridge, UK) was used at 0.5 μM. Cisplatin was used at 50 μM, and the caspase inhibitors Z-VAD(OMe)-FMK (SM Biochemicals LLC SMFMK001) and Q-VD-OPH (SM Biochemicals LLC SMPH001) at 50 μM. The inhibitors were dissolved in DMSO and the total concentration of DMSO in the culture medium did not exceed 1%.

### Generation of cells expressing constitutively active MKK6

U2OS cells were transfected with the constructs pcDNA4 TO (Invitrogen) and pcDNA6-MKK6DD using NanoJuice (Merc Millipore). MKK6DD is a constitutively active form of the specific p38 MAPK activator MKK6 with the activation loop residues Ser207 and Thr221 mutated to Asp. After transfection, cells were trypsinized, plated at dilutions of 1:4, 1:3 and 1:2, and grown in media containing 4 μg/ml Blasticidin S HCl (Invitrogen, A11139-03) and 35 μg/ml of Zeozin (Invitrogen, R250-01). Media was changed every 3 days and when colonies were large enough, they were picked and replated in a 24-well plate. Selected clones were amplified and tested for p38 MAPK activation. To induce MKK6DD expression, cells were plated at 2.5 × 10^5^ for a 6-well plate, 5 × 10^5^ for a 60-mm plate or 1.2 × 10^6^ for a 10-cm plate and when they reached 85% confluence, fresh media containing 1 μg/ml tetracycline (Sigma 87128-25 G) or the corresponding amount of ethanol (1:1000 dilution) was added. Tetracycline stocks were prepared in ethanol at 1 mg/ml and stored at −20 °C.

### Infection with shRNA-expressing lentivirus

To generate short hairpin RNAs (shRNA) in a lentivirus delivery system, 293 T cells (60% confluent) were transfected with a mix of the shRNA-encoding DNAs (5 μg) pLKO.1-shMAPK12 for p38γ (Sigma, Clone ID TRCN0000006145) or pLKO.1-shMAPK14 for p38α (Sigma, Clone ID TRCN0000000509), VSV-G (0.5 μg) and ∆89 packaging DNA (4.5 μg) using the calcium chloride method. After overnight, the media was replaced, and 48 h later, the media containing the virus was collected and passed through PVDF filters. For cell infection, the virus-containing media was diluted 1:10 in buffer containing 6 mg/l of of polybren, incubated for 10 min and then placed on the cell monolayer. The following day media was replaced, and 24 h later the cells started to be selected with puromycin (1 μg/ml) for 24–48 h. Selected cell populations were cultured in media with puromycin (1 μg/ml).

### Transfection with siRNAs

Cells were grown up to 60% confluence in 60 mm dishes and were transfected using Dharmafect transfection kit according to the manufacturer’s instruction with 5 μl of 25 μM siRNAs for c-Myc (ID 103828, Life Technologies, AM16708) and PPARGC1A (ID 108385, Life Technologies, AM51331) or scrambled (Ambion 4390843) as a control﻿, to the final concentration of 65 nM. Cells were plated 48 h later and led to recover for 16 h before proceeding with the experiment.

### Annexin V/PI staining

Cell death was assayed using FITC Annexin V kit (BD Pharmingen, 556547). The day before the experiment, 2 × 10^5^ cells/well were plated on a 6-well dish. After appropriate treatments, media was collected into 2 ml Eppendorf tube. The cell monolayer was washed once with PBD and 200 μl of trypsin was added. Cells were collected using the previously removed media. The whole population of dead and live cells was centrifuged for 5 min at 1000 rpm. Cells were wash once with 1x Binding buffer and re-suspended in 500 μl of Binding buffer in concentration 1 × 10^6^ cells/ml. 100 μl of the solution (1 × 10^5^ cells) was transferred into a new Eppendorf tube and 4.5 μl of FITC Annexin V were added. Staining was left for 20 min at RT in the dark, after which 450 μl of Binding buffer and 5 μl of PI solution was added. Samples were transferred to ice and analysed immediately.

### Proliferation assays

Cells were seeded at the density 3.5 × 10^4^ cells/well into 96 well plates 16 h prior to the treatments. Cell proliferation was analysed using the MTT Cell Growth Assay (Merck Millipore, CT02). The increased cell proliferation is presented as a fold change in absorbance of the treated cells with respect to the untreated controls measured at day 0.

### ATP measurement

ATP detection was assayed using an ATP measurement kit (Invitrogen, A22066). After treatments, cells were washed once with TBS, trypsinized and pelleted by centrifugation. Samples were immediately frozen on dry ice and stored at −80 °C. For ATP detection, frozen pellets containing 5–7 × 10^5^ cells were used. For sample preparation, 100 μl of boiling MQ water was added and the tubes were placed at 95 °C for 3 min and then on ice. To remove cellular fragments, samples were centrifuged for 5 min at 13000 rpm. Supernatants were collected and transferred to a new tube. Cellular lysates were diluted 3–5 times and levels of ATP were analysed according to the protocol provided by the manufacturer. Values were normalized to the cell numbers.

### Cellular respiration

Cells were plated 12 h prior to the experiment at 4 × 10^4^ cells into each well of a XF cell microplate in a total volume of 150 μl of DMEM containing 10 mM glucose and 2 mM glutamine. After 4 h, an additional 250 μl of media was added. Experiment was started by washing cells once with XF Cell Mito Stress Test Assay Medium and adding 500 μl/well of Assay Medium for the control and 500 μl/well of Assay Medium containing 1 μg/ml of tetracycline for MKK6 expression. After 12 h of induction measurement of the OCR begun. One h prior to the assay, the plate was placed in a 37 °C incubator without CO_2_. Meanwhile a cartridge port plate hydrated in an incubator without CO_2_ was prepared according to the manufacture’s recommendations. For the study, we used 1 μM oligomycin, 0.5 μM carbonyl cyanide m-chlorophenyl hydrazine (CCCP), 0.5 μM rotenone and antimycin. Oxygen consumption was measured using XFp Extracellular Flux Analyzer from Seahorse Bioscience and normalized towards protein quantity.

### GSH content

Total glutathione (GSSG + GSH) and reduced (GSH) glutathione were measured using the GSH/GSSG-Glo™ Assay (Promega). 5000 cells were plated into 96 well plates 18 h before the experiment. After treatment, media was removed and 50 μl of Total/Oxidized Glutathione Lysis Reagent was added and incubated 5 min on a plate shaker. Next 50 μl of Luciferin Generation Reagent was added into all wells and incubated at RT for 30 min. 100 μl of Luciferin Detection Reagent was added, incubated for 15 min and luminescence was measured. Data normalization was performed towards cell number.

### Mitochondria membrane potential

Each well of a 6 well dish received 2 ml of fresh complete medium containing 200 nM TMRE, and cells were incubated for 30 min in the dark at 37 °C. To remove the unbound probe, cells were washed once with PBS, trypsinized and resuspended in 500 μl of PBS containing aprotinin (1 μg/ml). Mitochondria membrane potential was assayed immediately by flow cytometry.

### Mitochondrial mass

Mitochondrial content was assayed by staining with Mitotracker Deep Red (Invitrogen). Each well of a 6 well dish, containing approximately 2 × 10^5^ cells received 2 ml of complete media with 200 nM Mitotracker Deep Red. After incubation for 30 min at 37 °C, the incubation media was removed and cells were washed once with PBS, trypsinized and resuspended in 500 μl of PBS containing aprotinin (1 μg/ml). Mitochondrial content was immediately assayed by flow cytometry.

### Mitochondrial and genomic DNA extraction and quantification

Cells were plated in triplicates in 32 mm plates and treated for the desired periods of time. After the treatment cells were trypsinized, washed in PBS and counted. 1 × 10^6^ cells were lysed in 515 μl of Lysis Solution (75 mM NaCl, 50 mM EDTA, 0.02% SDS, 0.4 mg/ml proteinase K) and incubated at 50 °C for 2 h. After adding one volume of isopropanol, samples were incubated at 4 °C for 2 h. Samples were centrifuged for 30 min at 8500 rpm at 4 °C. The pellet was washed with 70% cold ethanol, air dried, resuspended in 50 μM TE and stored at −20 °C. To perform RT-PCR, 2 μl of DNA (100 ng/μl), 5 μl of Syber Green, 5 μl of MQ water and 0.5 μl of Forward and Reverse primers were mixed. The ratio between genomic and mitochondrial DNA was calculated using primers towards Actin B and COXII, respectively.

### ROS detection

Two different probes were used to detect ROS production, 2′,7′-dichlorofluorescin diacetate (DCFH-DA) (Sigma, D6883), which detects general oxidation, and MitoSOX™ Red (ThermoFisher scientific, M36008), specific for mitochondrial ROS. One day prior to the treatments, 2 × 10^5^ cells were plated per well in a 6 well dish. To visualize ROS, DCFH-DA (final concentration 10 μM) or MitoSox (final concentration 5 μM) were added to the media for the last 30 min. After the incubation media was removed, cells were washed once with PBS, trypsinized and resuspended in 500 μl of PBS containing 1.4 μg/ml aprotinin. Analysis was performed immediately by flow cytometry.

### Lipid droplets

Cells were plated on 6 well plates and treated for the desired periods of time. A 0.5 µM solution of Nile Red in PBS was prepared and added to the cell monolayer for 10 min at RT. Cells were collected by trypsinization, re-suspended in 500 μl of PBS and analysed on a flow cytometer Ex/Em = 552/636 nm.

### Glucose consumption and glutamine consumption and lactate production

Cells (5 × 10^5^ per 60-mm plate) were plated in duplicates 18 h prior the experiment in media containing 10 mM glucose and 2 mM glutamine. The following day, cells for time 0 were collected and counted. Meantime fresh media containing 10 mM glucose and 2 mM glutamine were added was added to the other plates. At each time point, cells were trypsinized and counted, and media was collected and stored at −20 °C. D-glucose was determined by a modification of the hexokinase/glucose 6-phosphate dehydrogenase spectrophotometric assay^45^ by using commercial reagents. Glutamine was determined by conversion first to glutamate through glutaminase reaction and subsequently quantification of the glutamate concentration. Glutamate concentration was determined by its conversion to α-ketoglutarate through glutamate dehydrogenase in the presence of ADP and NAD^+^^45^. L-lactate in the media was determined by spectrophotometry^45^ based on the oxidation of lactate to pyruvate by lactate dehydrogenase and NAD^+^ in the presence of hydrazine. Production of NAD(P)H was measured at 340 nm in a Cobas-Mira Plus analyzer. The consumption of glucose and glutamine and the production of lactate in the media were corrected by time and the number of cells present during all the incubation period. Linear growth between each incubation time was assumed. All results were expressed as μmol × million cells^−1^ × h^−1^.

### Intracellular ammonia accumulation

Ammonia was detected using Ammonia Assay Kit (Abcam, ab83360). Cells (5 × 10^5^ per 60-mm plate) were plated in duplicates 18 h prior the experiment. After the treatment, cells were homogenized in the Assay Buffer to have final concentration 2 × 10^6^ cells/100 μl and centrifuged at 13000 × g for 10 min at 4 °C. 10 μl of supernatant was used for the analysis according to the manufacturer’s recommendations.

### mRNA expression analysis

Total RNA was extracted from cells using the RNA mini kit from Ambion according to the manufacturer’s instructions. To determine RNA concentrations, the absorbance at 260 nm was measured. cDNA was obtained from 1 μg of the purified RNA using SuperScript II Reverse Transcriptase (Invitrogen) in a final volume of 20 μl. For RT-PCR, 4 μl of cDNA (15 ng/reaction) were loaded in triplicates in a 96-well plate and 6 μl of the mix reaction were added. The plate was sealed and centrifuged for 1 min at 200  × g and run as follows: 50 °C for 2 min, 95 °C for 10 min, 40 cycles of denaturation at 95 °C for 15 s, annealing at 56 °C for 15 s, elongation at 72 °C for 60 s, and three final steps of 95 °C for 15 s, 60 °C for 2 min and 95 °C for 15 s. GAPDH was used as a reference and the ΔΔC(t) method was used to quantify gene expression. The primer sequences are presented in Table 1.

**Table 1:** Primers used for RT-PCR analysis.

Primer	Sequence
HK2_F	CCTCGGTTTCCCAACTCTG
HK2_R	CTGGTCAACCTTCTGCACTT
Glut1_F	CAGTATGTGGAGCAACTGTGT
Glut1_R	AAGGTCCGGCCTTTAGTCT
PFKFB3_F	CAGTTGTGGCCTCCAATATC
PFKFB3_R	GGCTTCATAGCAACTGATCC
GLS1_F	TGATCCTCGAAGAGAAGGTGG
GLS1_R	AGCAAATCTTCGAAGTGCAGAC
PGC1A_F	AGATCCTCTTCAAGATCCTGCT
PGC1A_R	ACGTGATCTCACATACAAGGG
ACTB_F	TCCTCCCTGGAGAAGAGCTA
ACTB_R	GAAGGAAGGCTGGAAGAGTG
COXII_F	ACGGCGGACTAATCTTCAAC
COXII_R	CGATTGTCAACGTCAAGGAG

### Immunoblotting

Approximately 40 μg of proteins were separated on 10 or 12% SDS-PAGE Laemmli gels, depending on the molecular weight of the protein of interest. A wet-blotting system (Bio Rad) was used for protein transfer to nitrocellulose membrane, which were stained with 0.1% Ponceau (in 5% acetic acid) to evaluate transfer efficiency. Membranes were blocked for 1 h at RT in 5% non-fatty milk (in PBS). Primary antibodies were diluted in PBS with 5% BSA and 0.1% Tween 20. The following antibodies were used at a concentration of 1:1000: p38α (Cell Signaling, 9218), P-p38 (Thr180/Tyr182) (Cell Signaling, 9211 S), P-MAPKAPK2 (Thr222) (Cell Signaling, 3044 S), P-Hsp27 (Ser82) (StressGene, SPA-523), P-PDHE1-A type I (Ser293) (Merk Millipore, ABS204), c-Myc (Abcam, ab32072), Tim-44 (BD, T14720), TFAM (Abcam, ab131607), VDAC-1 (Abcam, ab14734), P-CREB (Ser133) (Cell Signaling, #9191), P-eIF4E (Ser209) (Cell Signaling, #9741). The MKK6 rabbit antiserum has been previously described^46^, and the p38γ antibody was kindly provided by Ana Cuenda (CNB, Madrid). Tubulin (Sigma, T9026) was used at 1:5000 as a loading control. Secondary Alexa Fluor-conjugated antibodies were diluted 1:5000 in 1% non-fatty milk. Membranes were analysed using the Odyssey Infrared Imaging System (Li-Cor Biosciences).

### Incorporation of ^14^C into lipids and proteins

Cells were seeded at a density of 2.5 × 10^5^ cells/well (6-well plate), and 18 h later the medium was changed and cells were subjected to different treatments to activate or inhibit the p38 MAPK pathway. After 6 h, the medium was supplemented with trace amounts of [U^14^C] glucose or [U^14^C] glutamine (0.1 μCi/ml) and the cells were further incubated for 24 h. Experiments were conducted in triplicate. Cells were washed with PBS, collected using 200 μl 0.5% Triton X-100, and stored at −80 °C for 1–5 days. After thawing, 180 μl of the suspension was taken for the lipid and protein extraction. First 600 μl of chloroform/methanol (2:1) was added and vortexed for 1 min. Later the mixture was centrifuged 500  × g for 5 min. Lipid-rich chloroform phase was air dried overnight and resuspended in 200 μl of chloroform. Protein rich phase were washed with methanol and spun at 14,200  × g for 5 min and air dried overnight. Next day, the protein fraction was resuspended in 200 μl of guanidine hydrochloride 6 M and was incubated at 65 °C for 15 min. ^14^C incorporation was measured over 1 min per sample using a MicroBeta2 scintillation counter (PerkinElmer) and was normalized for protein concentration.

### Statistical analysis

All the statistics were performed using GraphPad Prism software using Student *t-*test unpaired two-tailed analysis: (****)p < 0.00001, (***)p < 0.0001, (**)p < 0.001, (*)p < 0.01.

## Electronic supplementary material

Supplementary Information
