# Supplementary material for: Induction of oxidative metabolism by the p38α/MK2 pathway
Source: Sci Rep. 2017 Sep 12;7:11367. doi: 10.1038/s41598-017-11309-7 (PMC5595987; doi:10.1038/s41598-017-11309-7)
Supplement: Supplementary file 1 — Supplementary Information [file 41598_2017_11309_MOESM1_ESM.pdf]

Supplementary Information

Induction of oxidative metabolism by the p38α/MK2 pathway

Natalia Trempolec, Juan Pablo Muñoz, Konstantin Slobodnyuk, Silvia Marin, Marta Cascante, Antonio Zorzano and Angel R. Nebreda

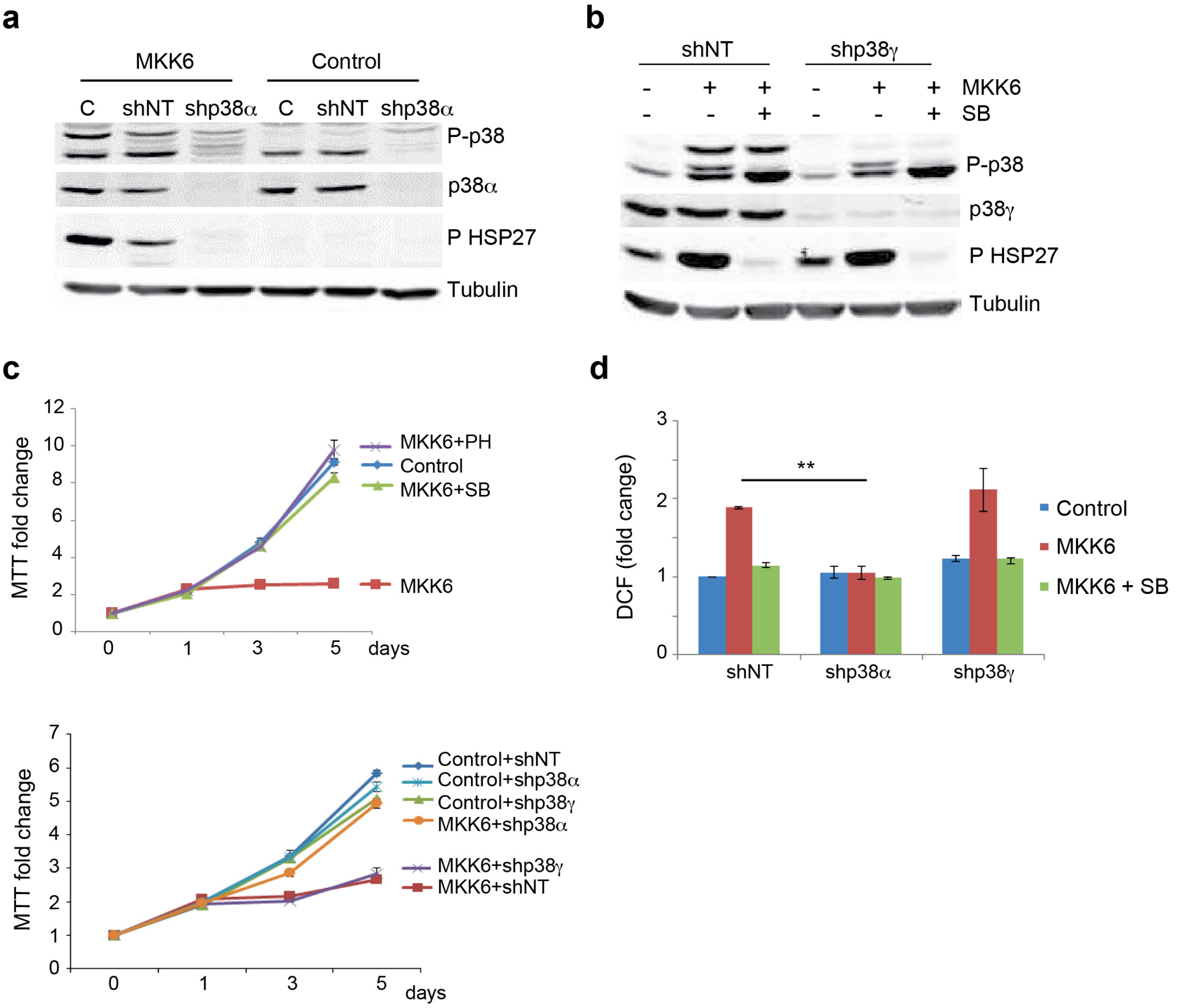

Supplementary Figure S1. MKK6 expression activates p38α and p38γ. U2OS cells expressing a Tet-regulated construct were either mock treated (Control) or treated with tetracycline for 24 h to induce the expression of constitutively active MKK6. (**a**) Cells were transfected with non-targeted (NT) or p38α shRNAs before treatment with tetracycline. Total cell lysates were analysed by immunoblotting using the indicated antibodies. (**b**) Cells were transfected with non-targeted (NT) or p38γ shRNAs, or incubated with the p38α inhibitor SB203580 (SB), as indicated, before treatment with tetracycline. Total cell lysates were analysed by immunoblotting using the indicated antibodies. (**c**) Proliferation of cells incubated with the p38α inhibitors SB or PH797804 (PH), or transfected with the indicated shRNAs, was measured at the indicated times after tetracycline addition using MTT. (**d**) Total ROS levels were analysed using the DCFH-DA probe. Values are presented as fold change of mean fluorescence of DCF versus the control (shNT) cells.

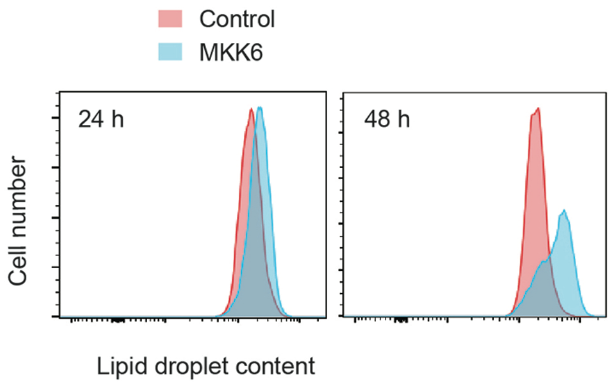

Supplementary Figure S2. MKK6 expression changes the lipid droplet content. U2OS cells expressing a Tet-regulated construct were either mock treated (Control) or treated with tetracycline for the indicated times to induce the expression of constitutively active MKK6. The lipid droplet content was analysed 24 and 48 h after MKK6 induction.

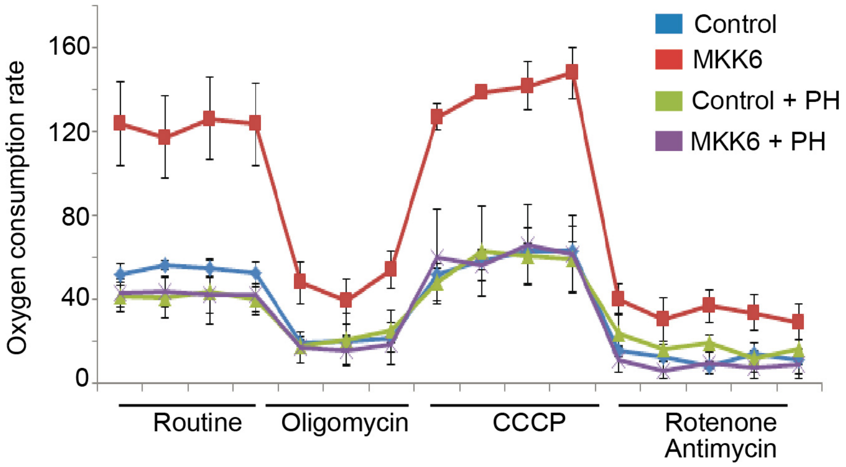

Supplementary Figure S3. MKK6 expression increases the oxygen consumption rate. U2OS cells expressing a Tet-regulated construct were either mock treated (control) or treated with tetracycline for 12 h to induce the expression of constitutively active MKK6. The oxygen consumption rate was analysed in the presence or absence of the p38α inhibitor PH797804 (PH). Results are presented as pmoles of O_2 consumed per 10^5 cells.

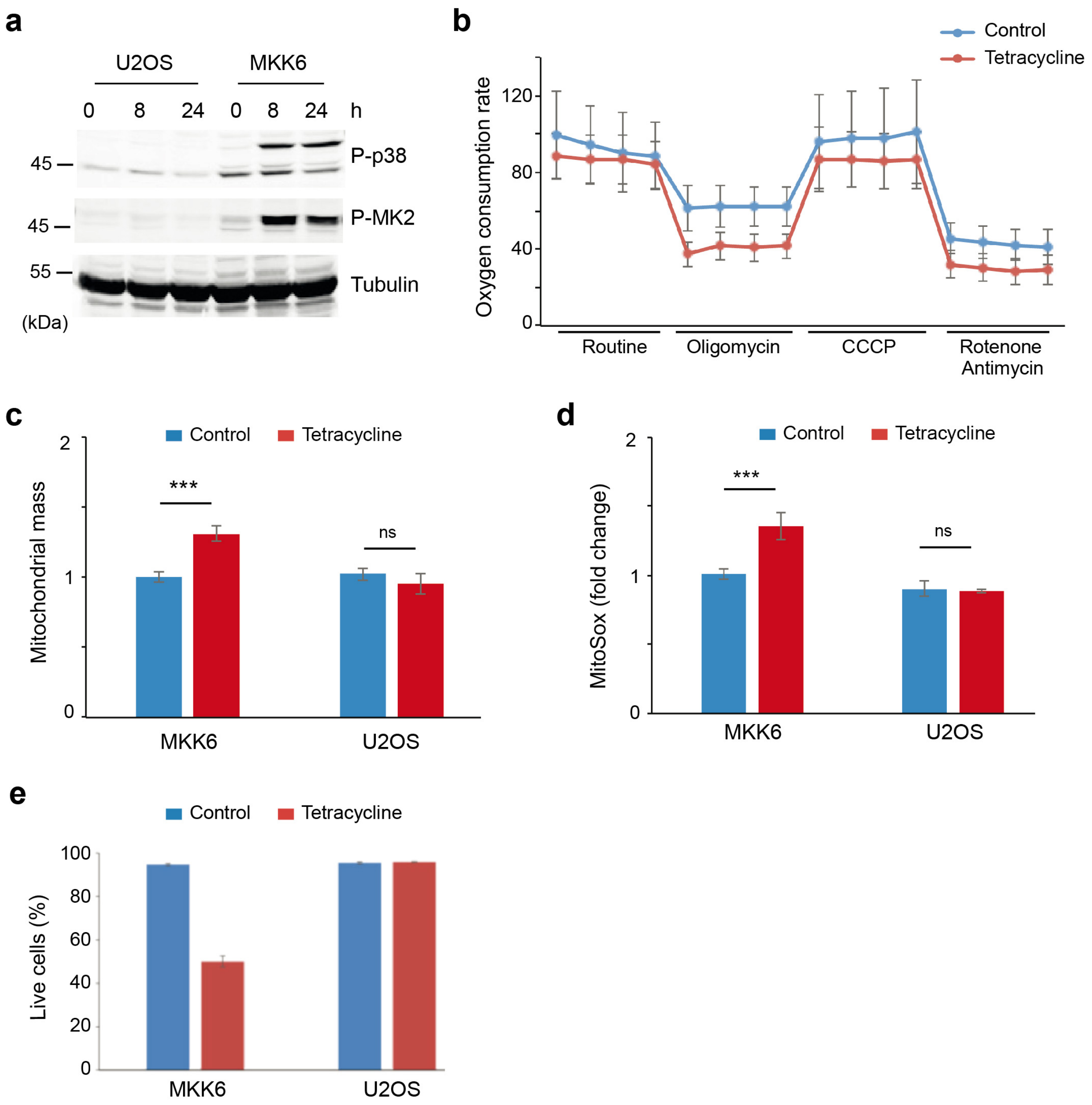

Supplementary Figure S4. Tetracycline alone induces neither p38α pathway activation nor mitochondrial changes. U2OS cells with a tet-inducible construct to express constitutively active MKK6 (MKK6) or U2OS cells that do not express the tet-regulated MKK6 construct (U2OS) were either left untreated (Control) or treated with tetracycline for the indicated times. (**a**) Total cell lysates were analysed by immunoblotting using the indicated antibodies. (**b**) Analysis of the oxygen consumption rate in U2OS cells that do not express the tet-regulated MKK6 construct either untreated or treated with tetracycline for 12 h. Results are presented as pmoles of O_2 consumed per 10^5 cells. (**c** and **d**) Mitochondrial biomass was analysed using MitoTracker Deep Red (**c**) and mitochondrial ROS was analyzed using MitoSOX (**d**) 24 h after the addition of tetracycline. (**e**) Cell survival was analyzed using AnnexinV/PI staining 3 days after the addition of tetracycline, and live cells were determined as the cell population Annexin V^- and PI^-. The experiment was repeated twice.

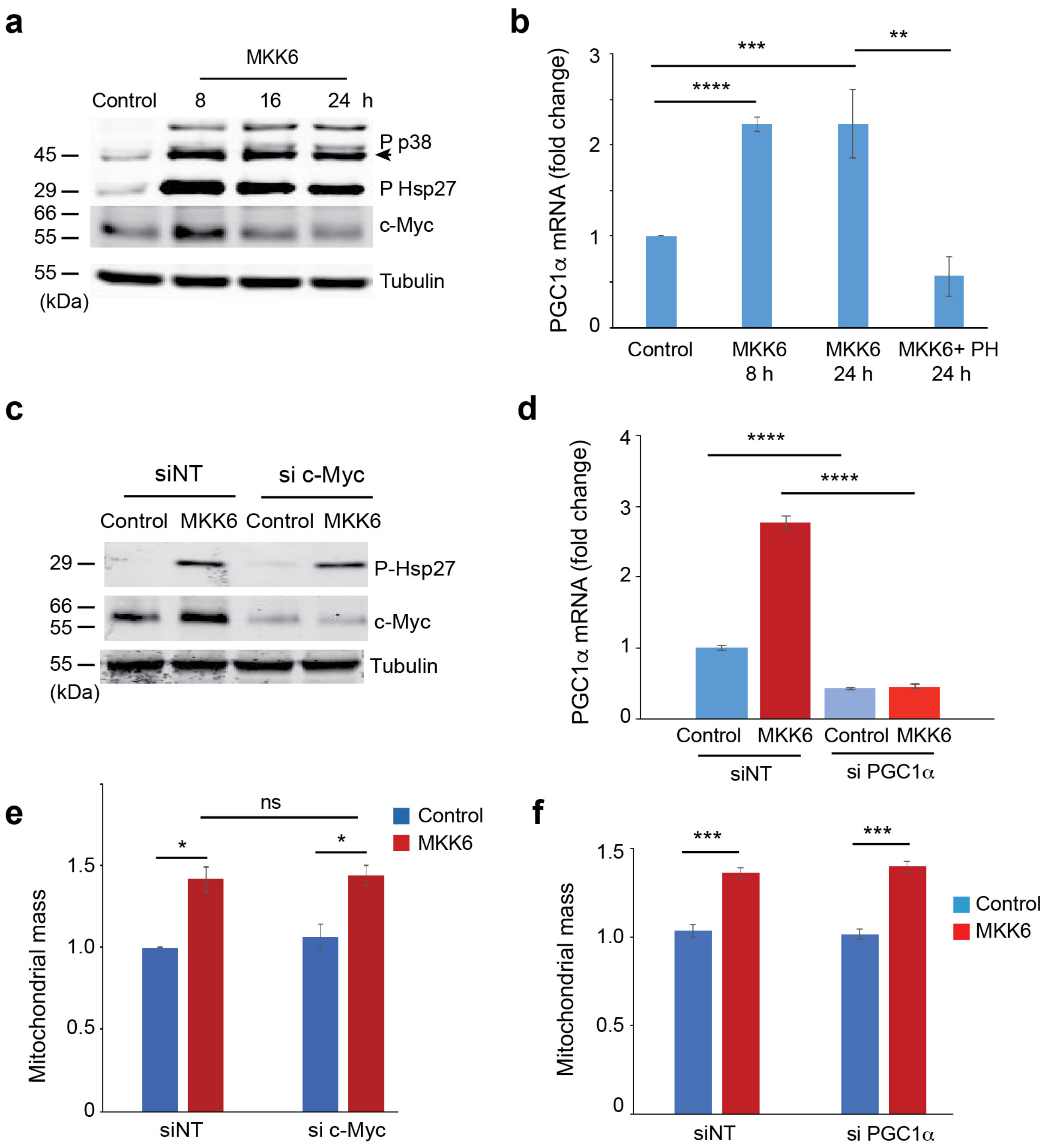

Supplementary Figure S5. c-Myc and PGC1α do not mediate the enhanced mitochondrial mass induced by MKK6 expression. U2OS cells expressing a Tet-regulated construct were either mock treated (Control) or treated with tetracycline for the indicated times to induce the expression of constitutively active MKK6. (**a**) Total lysates from control and cells expressing MKK6 were analysed by immunoblotting using the indicated antibodies. (**b**) Expression of the PGC1α mRNA was analysed at the indicated time points. (**c** and **e**) Cells were transfected with scrambled (siNT) or c-Myc siRNAs and 64 h later MKK6 expression was induced for 8 h. c-Myc expression was analysed by immunoblotting (**c**) and mitochondrial mass was detected using MitoTracker Deep Red (**e**). (**d** and **f**) Cells were transfected with scrambled (siNT) or PGC1α siRNAs and 64 h later MKK6 expression was induced for 8 h. Expression of PGC1α mRNA was determined by qRT-PCR (**d**), and mitochondrial mass was detected using MitoTracker Deep Red (**f**).

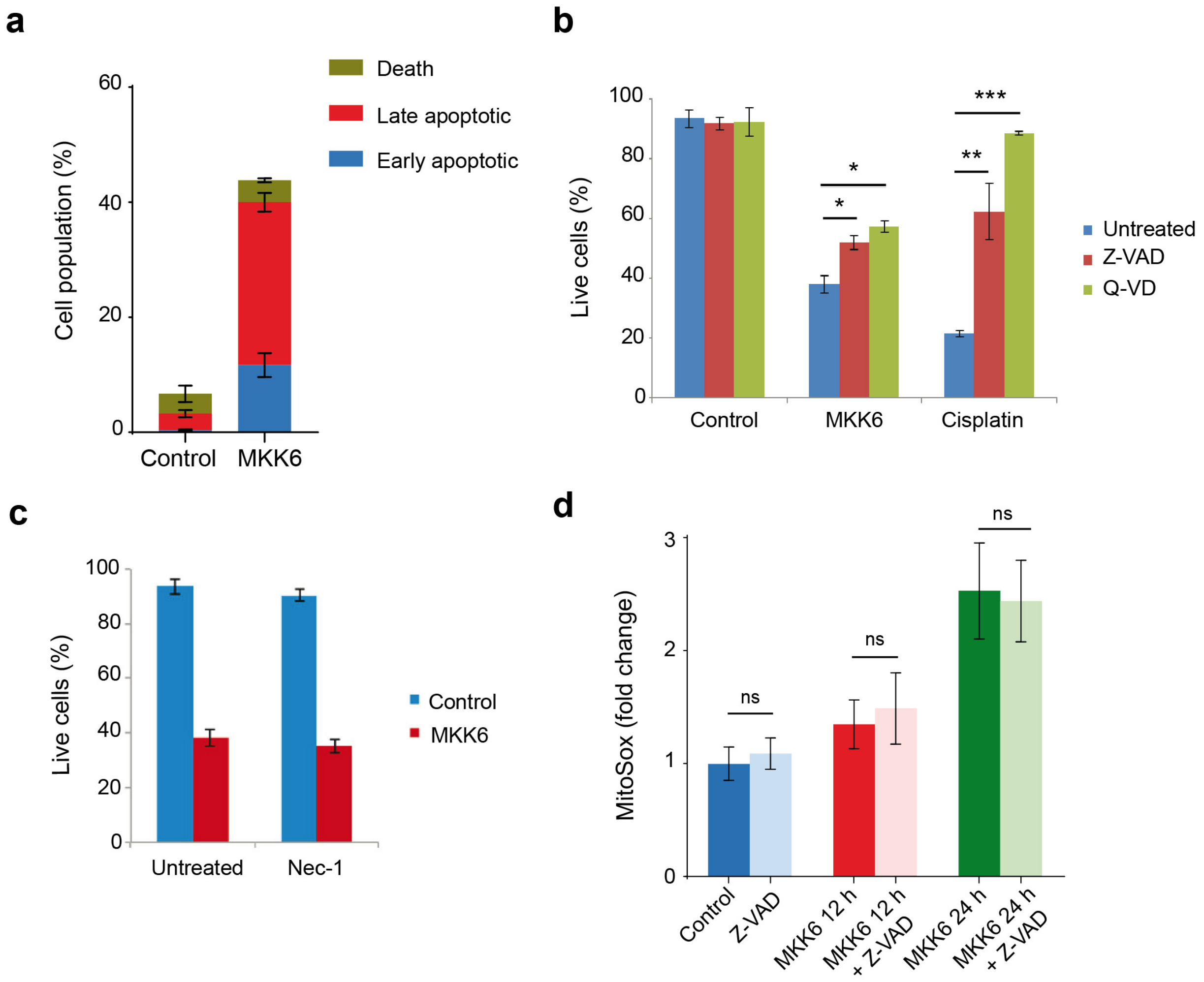

Supplementary Figure S6. Cell death induced by MKK6 expression. U2OS cells expressing a Tet-regulated construct were either mock treated (Control) or treated with tetracycline for three days to induce the expression of constitutively active MKK6. (**a**) Cell death was analysed using Annexin V/PI staining. Early apoptotic refers to Annexin V^+ and PI^- cells, Late apoptotic to the Annexin V^+ and PI^+ cells, and Death indicates the cell population that was Annexin V^- and PI^+. (**b**) Cells were incubated with the indicated caspase inhibitors and then were either treated with tetracycline to induce MKK6 expression or treated with cisplatin. Cell survival was assayed using Annexin V/PI staining, and live cells were determined as the cell population Annexin V^- and PI^-. (**c**) Cells were incubated with the necrosis inhibitor Nec-1 (10 µM) and then MKK6 expression was induced. Cell survival was assayed using Annexin V/PI staining, and live cells were determined as the cell population Annexin V^- and PI^-. (**d**) Mitochondrial ROS levels in control and MKK6 expressing cells either mock-treated or treated with Z-VAD were evaluated at the indicated times using the MitoSox probe. Values are represented as fold change in the mean fluoresce of MitoSox normalized towards control cells.

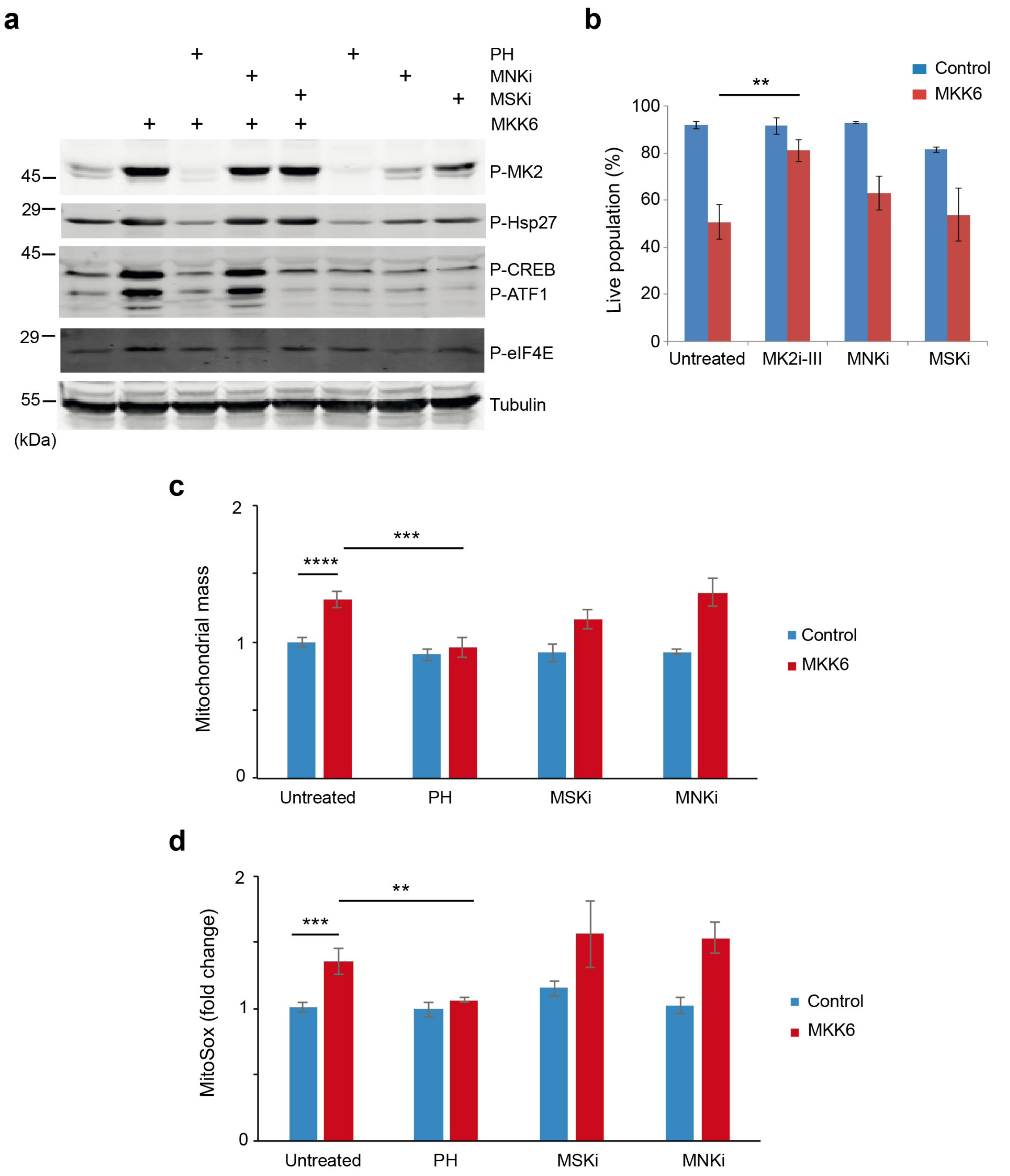

Supplementary Figure S7. MSK and MNK inhibition does not affect MKK6-triggered mitochondrial changes. U2OS cells expressing a Tet-regulated construct were either mock treated (Control) or treated with tetracycline to induce the expression of constitutively active MKK6. (**a**) Cells were incubated with the p38α inhibitor PH797804 (PH), the MSK inhibitor SB747651 (MSKi) or the MNK inhibitor CGP-57380 (MNKi) and then MKK6 expression was induced for 8 h. Total cell lysates were analysed by immunoblotting using the indicated antibodies. (**b**) Cells were incubated with the MK2 inhibitor III (MK2i-III) or the MSK and MNK inhibitors indicated in (**a**), and then MKK6 expression was induced for 2 days. Cell survival was assayed using Annexin V/PI staining, and live cells were determined as Annexin V^- and PI^- cell population. (**c** and **d**) Cells were incubated with the p38α, MSK or MNK inhibitors indicated in (**a**), and then MKK6 expression was induced for 24 h. Mitochondrial biomass was analysed using MitoTracker Deep Red (**c**), and Mitochondrial ROS was analysed using MitoSOX (**d**).

Figure 1a

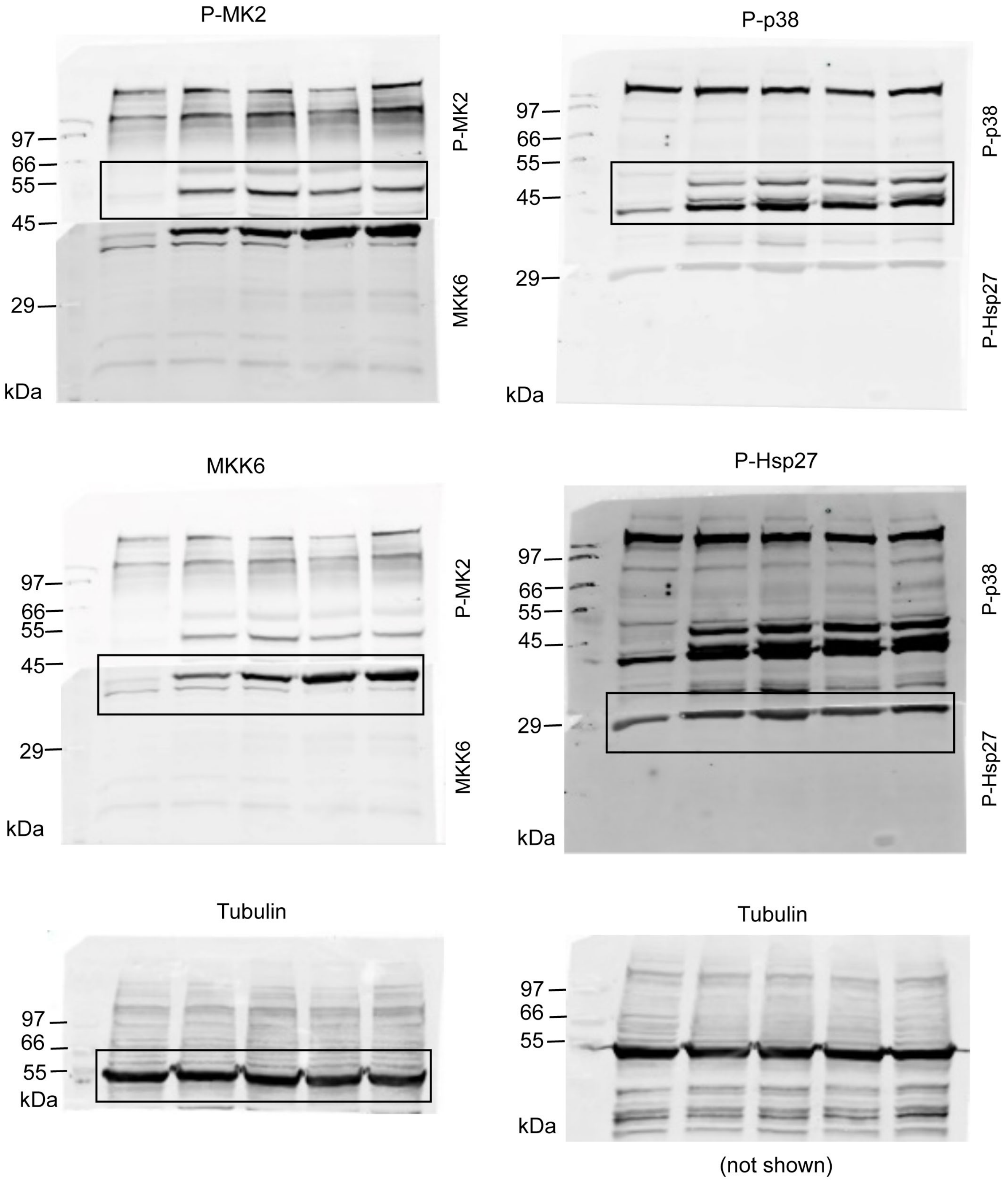

Supplementary Figure S8. Uncropped immunoblots of the indicated figures. For Figure 1a, two membranes were cut at the 45 kDa marker (left) and 29 kDa marker (right), and then the upper and lower parts were independently incubated with the indicated antibodies. After incubation with the secondary antibodies, the two parts of each membrane were put together for scanning. Both membranes were also incubated with Tubulin antibodies.

Figure 2c

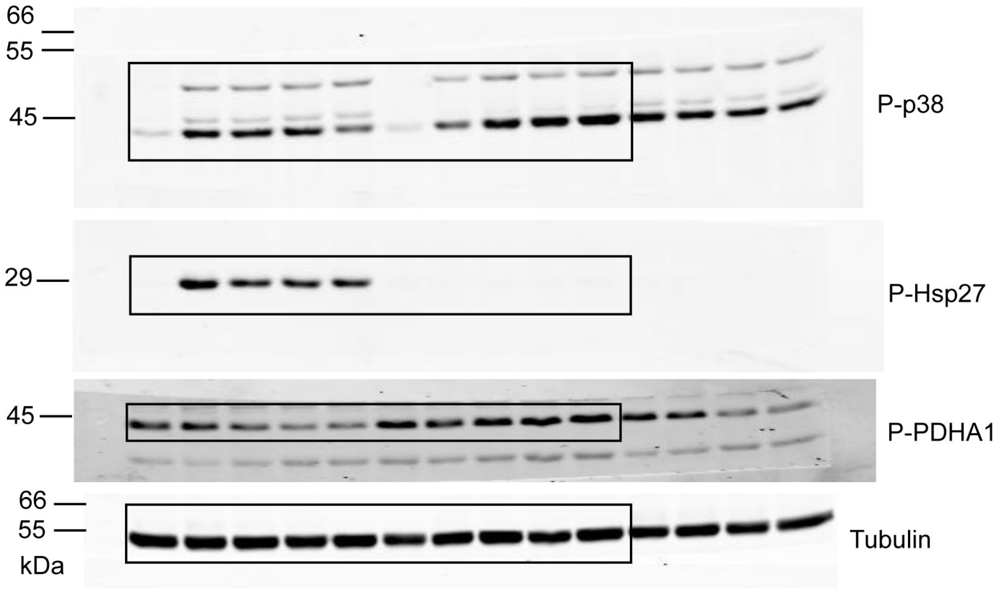

Figure 3b

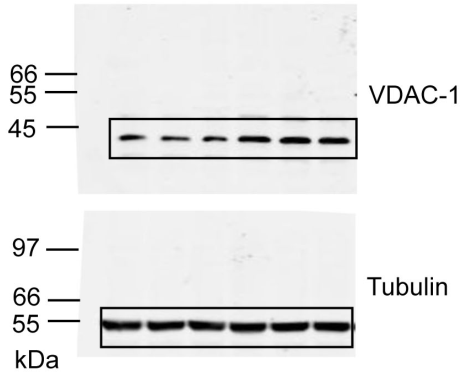

Figure 3c

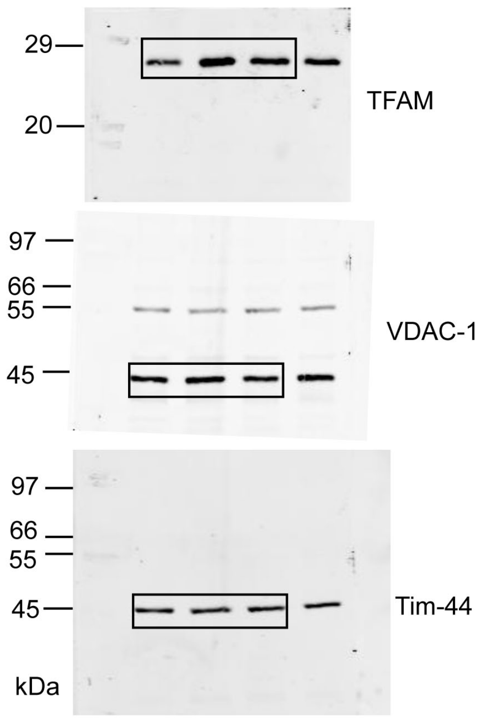

Figure 6d

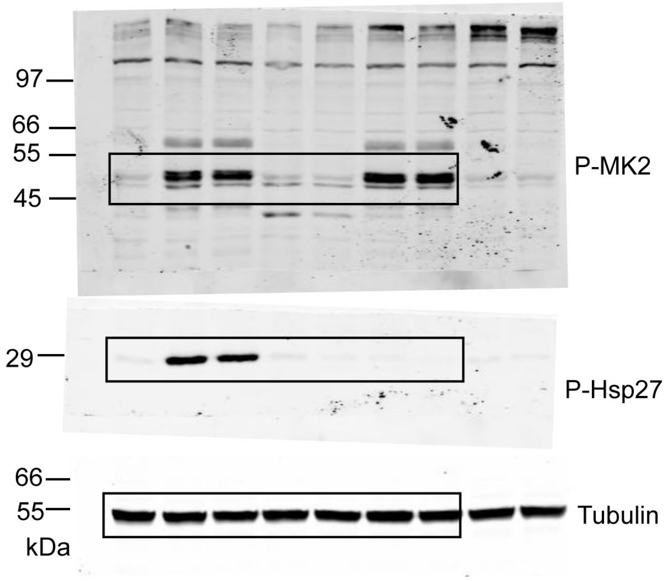

Supplementary Figure S8 (cont.). Uncropped immunoblots of the indicated figures.
